# COVID-19 and cardiovascular outcomes in patients with pre-existing hypertension

**DOI:** 10.1038/s41371-026-01147-4

**Published:** 2026-04-09

**Authors:** Roham Hadidchi, Suhani Pahuja, Shiv Mehrotra-Varma, William Zhao, Ryan C. Lee, Sonya Henry, Tim Q. Duong

**Affiliations:** Department of Radiology, Albert Einstein College of Medicine and Montefiore Health System, Bronx, NY USA

**Keywords:** Risk factors, Medical research

## Abstract

Patients with hypertension have worse acute COVID-19 outcomes, but the long-term effects of SARS-CoV-2 infection is unclear. We conducted a retrospective cohort study of adults with hypertension and no prior cardiovascular events in the Montefiore Health System, comparing those with and without COVID-19 over up to 4.5 years post-infection. Outcomes included first-time myocardial infarction (MI), heart failure (HF), stroke, all-cause mortality, and major adverse cardiovascular events (MACE). Multivariate regression and inverse-probability weighting adjusted for demographics, comorbidities, socioeconomic status, and COVID-19 vaccination. Adjusted hazard ratios (HRs) with 95% confidence intervals were calculated. Sub-analyses examined hypertension stage and acute COVID-19 blood biomarkers in relation to outcomes. Among 75,180 hypertensive patients, hospitalized COVID-19 was associated with increased risk of first-time MI (adjusted HR = 1.40 [1.21–1.63]), HF (1.59 [1.45–1.75]), stroke (1.35 [1.17–1.57]), all-cause mortality (2.51 [2.17–2.90]), and MACE (1.65 [1.54–1.77]) compared to COVID-negative individuals. Non-hospitalized COVID-19 patients had elevated risks of HF (1.17 [1.06–1.30]) and MACE (1.14 [1.05–1.23]). Hospitalized COVID-19 was associated with an increase in MACE risk by 75% in those with normal blood pressure, and by 126% and 148% in those with elevated blood pressure and stage 1 hypertension, respectively. Abnormal C-reactive protein, creatinine, lactate dehydrogenase, D-dimer, hemoglobin, and neutrophil-to-lymphocyte ratio predicted higher MACE risk. COVID-19, irrespective of disease severity, puts hypertensive patients at greater risks of worse cardiovascular outcomes, especially those with more advanced hypertension. These findings underscore the importance of long-term cardiovascular monitoring in this vulnerable population.

## Introduction

Hypertension is the most prevalent cardiovascular risk factor worldwide, affecting nearly half of adults in the United States alone [1]. Chronic elevations in blood pressure accelerate arterial stiffening, promote atherosclerotic plaque development, and contribute to left ventricular hypertrophy, pathophysiological changes that increase the likelihood of myocardial infarction (MI), heart failure (HF), and stroke [2–5]. Although the cardiovascular consequences of hypertension are well established, the emergence of COVID-19 has introduced new complexity into the long-term management of this condition [6, 7].

Many studies have reported that COVID-19 survivors are at heightened risk for new-onset hypertension [8–12], MI, HF, and stroke [13–16]. However, few studies have focused specifically on how SARS-CoV-2 infection impacts long-term cardiovascular outcomes in patients already diagnosed with hypertension. Mechanistically, SARS-CoV-2 infection may exacerbate hypertension-related cardiovascular risk through direct and indirect pathways. Viral engagement of angiotensin-converting enzyme 2 (ACE2) receptors downregulates their expression, leading to increased angiotensin II signaling and its downstream effects, vasoconstriction, inflammation, oxidative stress, and fibrosis [8]. Additional injury to endothelial cells and microvascular structures further promotes thrombogenesis and vascular dysfunction [17, 18]. In combination, these processes could accelerate cardiovascular deterioration in hypertensive individuals.

Given the high global burden of hypertension and the widespread impact of COVID-19, understanding how these conditions interact to influence long-term cardiovascular outcomes is a critical public health priority. In this study, we examined the association between COVID-19 and all-cause mortality, as well as first-time MI, HF, and stroke, in a large cohort of patients with pre-existing hypertension, followed for up to 4.5 years post-infection. We further assessed the role of baseline hypertension severity, socioeconomic status, and acute-phase biomarkers in shaping long-term cardiovascular risk in this vulnerable population. A more refined understanding of these interrelationships will inform future hypotheses and guide prospective research into preventive and therapeutic strategies for COVID-19 survivors with hypertension.

## Methods

### Data sources

This retrospective cohort study was approved by the Einstein-Montefiore Institutional Review Board with an exemption for informed consent (#2021–13658). Electronic health records (EHR) came from the Montefiore Health System (January 1, 2016–August 17, 2024), which consists of multiple hospitals and outpatient clinics in the Bronx and its environs. EHR data were extracted using Observational Medical Outcomes Partnership (OMOP) common data model as previously described [19–46]. A team of data scientists and engineers created, maintained, and validated data extraction. To ensure data quality, this team routinely performed manual chart review of all relevant variables on subsets of patients.

As retrospective studies are susceptible to patient selection biases, confounders, and competing risks, we corroborated our findings using inverse probability weighting (IPW) and multivariate regression to account for all major confounders. In addition, we also performed sensitivity analyses, including but not limited to, estimates of the magnitude of unknown confounders needed to invalidate our findings. With a large and diverse patient population, these findings are likely generalizable, although additional studies are needed to achieve broader generalizability.

### Study cohort

Inclusion criteria were adults (≥21 years old) with hypertension at index date but without prior history of outcomes (MI, HF, or stroke). Hypertension was defined meeting any of the following three criteria: 1) at least three BP measurements within the year prior to index date that all showed systolic BP ≥ 120 or diastolic BP ≥ 80, 2) ICD-10 diagnosis of hypertension at or prior to index date (I10 − I15), 3) documented use of antihypertensive medications at or prior to index date (see Appendix 1 for list) [12]. Patients classified as COVID+ were those tested positive by polymerase chain reaction (PCR) at least once and index date was defined as date of first positive test. COVID− controls consisted of those who tested negative and never tested positive and index date was defined as date of first negative test. Patients who did not return to our health system 30 days or more after the index date (lost to follow-up) were excluded. Patients who experienced outcomes (MI, HF, stroke, or mortality) within 30 days of index date were also excluded. These exclusions were applied to reduce misclassification of acute-phase events that may not represent long-term cardiovascular risk. However, we recognize this may introduce selection bias by removing sicker individuals and performed a sensitivity analysis without these 30-day exclusions.

### Variables

Demographic data included age at index date, sex, race, and ethnicity. Pre-existing comorbidities at index date included artery disease (CAD), type-2 diabetes (T2DM), chronic obstructive pulmonary disease (COPD), asthma, chronic kidney disease (CKD), liver disease, obesity, and tobacco use as defined by ICD-10 codes. Those who had no diagnosis of comorbidities or outcomes in the EHR were assumed to not have experienced the outcome or had the comorbidity.

BP classifications were based on average systolic and diastolic measurements in the year prior to index. Normal BP was defined as systolic <120 mmHg and diastolic <80 mmHg. Elevated BP was defined as systolic 120–129 mmHg and diastolic <80 mmHg; stage 1 hypertension as systolic 130–139 mmHg or diastolic 80–89 mmHg; and stage 2 hypertension as systolic ≥140 mmHg or diastolic ≥90 mmHg, in accordance with American College of Cardiology/American Heart Association guidelines [47]. A separate category was created for those with no BP measurements in the year preceding index date.

We collected data on vaccination for COVID-19 and considered patients vaccinated if they had received at least one dose prior to index date. Vaccination data were sourced from the New York State Immunization Information System (including New York City data), patient self-report, Care Everywhere data shared across Epic organizations, and vaccinations administered within the Montefiore Health System. This allowed for capture of both in-system and external COVID-19 vaccinations.

Socioeconomic data, including median annual household income of Zone Improvement Plan (ZIP) code, insurance, and unmet social needs, were also extracted. Insurance was obtained via the EHR and categorized as private, Medicare, Medicaid, and uninsured. Voluntary unmet social needs screener included 8 categories: housing, food insecurity, utilities, health transportation, medications, child or elderly care, legal services, and family safety. Patients were categorized into three groups: at least one unmet social need, no unmet social needs, and unknowns (screener not offered or did not complete).

To assess the severity of SARS-CoV-2 infection, COVID+ patients were stratified based on hospitalization during the acute phase of the infection. Among the hospitalized COVID+ cohort, the following biomarkers at time of infection were collected: neutrophil-to-lymphocyte ratio (NLR), hemoglobin (g/dL), platelets (110 × 10^9^ cells/L), ferritin (μg/L), D-dimer (μg/mL), creatinine (mg/dL), C-reactive protein (mg/dL), lactate dehydrogenase (U/L), aspartate aminotransferase (U/L), and temperature (°C) [48, 49].

### Outcome events

Outcome events included all-cause mortality, MI, HF, stroke, and MACE (defined as a composite of the four individual outcomes) recorded in the EHR > 30 days to up to 4.5 years after index date. Follow-up time was calculated in months from the index date to either the date of first diagnosis (for patients who developed the outcome) or to the date of death or last recorded visit (for patients who did not develop the outcome) up to August 17, 2024.

### Statistical analysis

Python (version 3.8.19) and R (version 4.4.0) were used for data processing and statistical analysis and *p*-values less than 0.05 were considered statistically significant. For group comparison of categorical variables, the χ2 test was used and for group comparison of continuous variables, the independent t-test was used. Standardized mean differences (SMDs) were also calculated to assess the magnitude of between-group differences, with values > 0.10 considered potentially meaningful. Risk of outcomes was assessed using Cox proportional hazards models for all-cause mortality and MACE and Fine-Gray subdistribution competing risk regression models for MI, HF, and ischemic or hemorrhagic stroke to account for mortality as a competing risk [50]. We evaluated the proportional hazards assumption using Schoenfeld residuals and time-by-covariate interaction terms and found no evidence of violation. Multivariate regression and IPW were used to adjust for baseline age, sex, race, ethnicity, comorbidities, stage of hypertension, insurance status, tertile of ZIP code median income, presence of unmet social needs, and SARS-CoV-2 vaccination status. To assess whether the association between SARS-CoV-2 infection and MACE differs by baseline stage of hypertension, hazard ratios (HR) were computed separately for patients with normal BP, elevated BP, stage 1 hypertension, and stage 2 hypertension and compared using interaction terms, adjusting for the same set of covariates using IPW. Among patients hospitalized with COVID-19, biomarkers at time of COVID-19 hospitalization were assessed and analyzed with respect to risk of MACE, adjusting for the same set of covariates using IPW. To assess the potential influence of unmeasured and residual confounders, we used an array approach, which explores the effect of a hypothetical binary residual confounder to assess the extent of confounder variable prevalence imbalance and effect size necessary to fully explain the observed effect estimate [51].

## Results

**Fig. 1:**
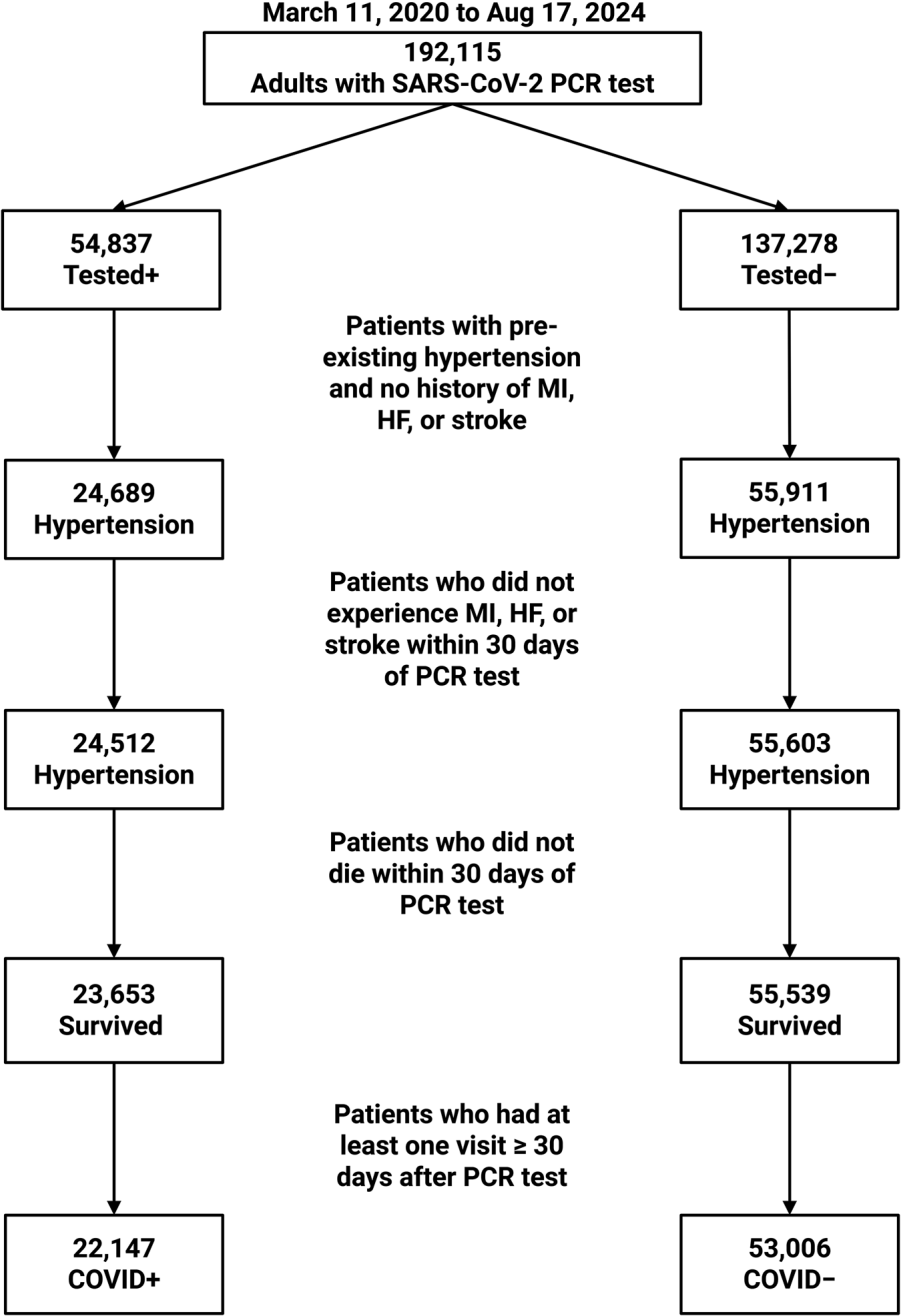
Patient selection flowchart. PCR, polymerase chain reaction. MI, myocardial infarction. HF, heart failure.

Figure 1 shows the patient selection flowchart. From March 11, 2020 to August 17, 2024, 192,115 adults (≥21 years old) had a SARS-CoV-2 PCR test performed for suspicion of respiratory infection. After only including survivors with pre-existing hypertension but no history of MI, HF, or stroke, 22,147 patients who were COVID+ and 53,006 patients who were COVID− were identified.

Table 1 presents the baseline demographic and clinical characteristics of patients with and without COVID-19. Compared to COVID– patients, those with COVID-19 had shorter mean follow-up (24.53 vs 27.33 months; SMD = 0.20), were slightly older (55.36 vs 54.68 years; SMD = 0.042) and had a similar sex distribution (female: 63.95% vs 62.37%; SMD = 0.033). Racial and ethnic distributions were also similar across groups (all SMDs <0.03).

**Table 1:** Characteristics of patients with pre-existing hypertension but no history of myocardial infarction, heart failure, or stroke with and without COVID-19. SD, standard deviation.

	COVID + (n = 22147)	COVID– (n = 53006)	*p*-value	SMD
Follow Up Time (Months), mean ± SD	24.53 ± 13.73	27.33 ± 14.27	**<0.005**	0.20
Age at Index Date (Years), mean ± SD	55.36 ± 16.74	54.68 ± 15.64	**<0.005**	0.042
Female, n (%)	14163 (63.95%)	33058 (62.37%)	**<0.005**	0.033
**Race and Ethnicity, n (%)**				
Non-Hispanic White	2053 (9.27%)	5345 (10.08%)	**<0.005**	0.028
Black	7970 (35.99%)	18331 (34.58%)	**<0.005**	0.029
Asian	1025 (4.63%)	2153 (4.06%)	**<0.005**	0.028
Other Race	11099 (50.12%)	27177 (51.27%)	**<0.005**	0.023
Hispanic	9341 (42.18%)	21915 (41.34%)	**0.035**	0.017
**Blood Pressure (mm Hg), mean ± SD**				
Systolic Blood Pressure	133.01 ± 13.78	133.91 ± 14.21	**<0.005**	0.065
Diastolic Blood Pressure	78.75 ± 8.42	80.02 ± 8.21	**<0.005**	0.15
**Stage of Hypertension, n (%)**				
No Blood Pressure Measurements Available	3965 (17.90%)	11083 (20.91%)	**<0.005**	0.076
Normal	2215 (10.00%)	3838 (7.24%)	**<0.005**	0.098
Elevated	3944 (17.81%)	8351 (15.75%)	**<0.005**	0.055
Stage 1 Hypertension	6780 (30.61%)	16611 (31.34%)	0.052	0.016
Stage 2 Hypertension	5243 (23.67%)	13123 (24.76%)	**<0.005**	0.025
**Definition of Hypertension Met, n (%)**				
Blood Pressure Measurements	15967 (72.10%)	38085 (71.85%)	0.50	0.0055
Antihypertensive Use	13361 (60.33%)	29535 (55.72%)	**<0.005**	0.093
ICD-10 Code	15287 (69.03%)	33288 (62.80%)	**<0.005**	0.13
**Pre-Existing Comorbidities, n (%)**				
Coronary Artery Disease	2092 (9.45%)	3222 (6.08%)	**<0.005**	0.13
Type-2 Diabetes	7512 (33.92%)	14308 (26.99%)	**<0.005**	0.15
COPD	993 (4.48%)	1214 (2.29%)	**<0.005**	0.12
Asthma	5101 (23.03%)	9210 (17.38%)	**<0.005**	0.14
Chronic Kidney Disease	3536 (15.97%)	5106 (9.63%)	**<0.005**	0.19
Liver Disease	2126 (9.60%)	4027 (7.60%)	**<0.005**	0.071
Obesity	13040 (58.88%)	28537 (53.84%)	**<0.005**	0.10
Tobacco Use	7642 (34.51%)	18757 (35.39%)	**0.022**	0.018
**Insurance, n (%)**				
Medicaid	7573 (34.19%)	19259 (36.33%)	**<0.005**	0.045
Medicare	4321 (19.51%)	9249 (17.45%)	**<0.005**	0.053
Private	8581 (38.75%)	21185 (39.97%)	**<0.005**	0.025
Uninsured	1672 (7.55%)	3313 (6.25%)	**<0.005**	0.051
**Income Group, n (%)**				
Lower Third (≤$42,639/year)	8942 (40.38%)	21668 (40.88%)	0.20	0.010
Middle Third ($42,834/year–$61,272/year)	6536 (29.51%)	15009 (28.32%)	**<0.005**	0.026
Top Third (≥$61,414/year)	6669 (30.11%)	16329 (30.81%)	0.061	0.015
**Unmet Social Needs, n (%)**				
At Least One Unmet Social Need	2080 (9.39%)	4258 (8.03%)	**<0.005**	0.048
No Unmet Social Needs	6062 (27.37%)	13405 (25.29%)	**<0.005**	0.047
Status Unknown	14005 (63.24%)	35343 (66.68%)	**<0.005**	0.072
**Hospitalized Due to COVID-19, n (%)**	6461 (29.17%)	0 (0.00%)	**<0.005**	0.91
**Vaccinated for SARS-CoV-2, n (%)**	8645 (39.03%)	15940 (30.07%)	**<0.005**	0.19
**Outcomes, n (%)**				
All-Cause Mortality	488 (2.20%)	560 (1.06%)	**<0.005**	0.091
Myocardial Infarction	464 (2.10%)	859 (1.62%)	**<0.005**	0.035
Heart Failure	1178 (5.32%)	1967 (3.71%)	**<0.005**	0.078
Ischemic or Hemorrhagic Stroke	459 (2.07%)	882 (1.66%)	**<0.005**	0.030
Major Adverse Cardiovascular Events	2088 (9.43%)	3544 (6.69%)	**<0.005**	0.10

*SMD*, standardized mean difference; *COPD*, chronic obstructive pulmonary disease.

Bold cases indicate statistical significance.

Hypertension stages were similarly distributed between COVID+ and COVID– patients (all SMDs <0.1). Among COVID+ patients, 10.0% had normal blood pressure, 17.8% had elevated BP, 30.6% had stage 1 hypertension, and 23.7% had stage 2 hypertension. Comparable proportions were observed in the COVID– group: 7.2% normal, 15.8% elevated, 31.3% stage 1, and 24.8% stage 2. The prevalence of hypertension, as defined by BP readings (72.1% vs 71.9%; SMD = 0.0055), was comparable, though COVID+ patients were more likely to meet hypertension criteria via ICD-10 codes (69.0% vs 62.8%; SMD = 0.13) and use of antihypertensives (60.3% vs 55.7%; SMD = 0.093).

Cardiometabolic and respiratory comorbidities were more common among COVID+ patients, including CAD (9.5% vs 6.1%; SMD = 0.13), T2DM (33.9% vs 27.0%; SMD = 0.15), COPD (4.5% vs 2.3%; SMD = 0.12), asthma (23.0% vs 17.4%; SMD = 0.14), and CKD (16.0% vs 9.6%; SMD = 0.19). Obesity was more prevalent in COVID-19 patients (58.9% vs 53.8%; SMD = 0.10), while tobacco use was similar (34.5% vs 35.4%; SMD = 0.018).

Sociodemographic differences were modest: COVID+ patients were equally likely to be insured privately (38.8% vs 40.0%; SMD = 0.025), on Medicare (19.5% vs 17.5%; SMD = 0.053), and had similar income distribution (all SMDs <0.03). A similar proportion had at least one unmet social need (9.4% vs 8.0%; SMD = 0.048). COVID+ patients were more frequently vaccinated against SARS-CoV-2 (39.0% vs 30.1%; SMD = 0.19) and had a 29.2% hospitalization rate due to COVID-19.

Incidence of outcomes (unadjusted for observation time and confounders) was higher among COVID+ as compared to COVID– patients (all *p* < 0.005 though SMDs ≤0.1).

Table 2A shows the Cox proportional and Fine-Gray subdistribution adjusted HR for COVID-19 and risk of cardiovascular outcomes. After adjusting for baseline age, sex, race, ethnicity, comorbidities, stage of hypertension, insurance status, tertile of ZIP code median income, presence of unmet social needs, and SARS-CoV-2 vaccination status, patients hospitalized for COVID-19 were more likely to experience all-cause mortality (adjusted HR = 2.51 [2.17, 2.90]), MI (1.40 [1.21, 1.63]), HF (1.59 [1.45, 1.75]), stroke (1.35 [1.17, 1.57]), and MACE (1.65 [1.54, 1.77]) compared to the COVID– group. Non-hospitalized COVID+ patients were at higher risk of HF and MACE compared to COVID– controls. Supplementary Table 1 shows the full results of multivariate models with all covariates. For the outcome of MACE, those who were Black or Hispanic, or had comorbidities were at higher risk. Additionally, those with at least one unmet social need, and those on Medicare or Medicaid were more likely to experience MACE.

**Table 2:** Cox proportional (all-cause mortality and major adverse cardiovascular events) and Fine-Gray subdistribution (myocardial infarction, heart failure, and ischemic or hemorrhagic stroke) adjusted hazard ratios (HR) for different outcomes grouped by COVID-19 status (COVID+ hospitalized and COVID+ non-hospitalized vs. COVID–).

	A) Multivariate Regression			
Outcome	COVID+ Hospitalized vs COVID–		COVID+ Non-Hospitalized vs COVID–	
	Adjusted HR [95% CI]	*p*-value	Adjusted HR [95% CI]	*p*-value
All-Cause Mortality	2.51 [2.17, 2.90]	**<0.005**	1.16 [0.96, 1.39]	0.12
Myocardial Infarction	1.40 [1.21, 1.63]	**<0.005**	1.08 [0.92, 1.26]	0.34
Heart Failure	1.59 [1.45, 1.75]	**<0.005**	1.17 [1.06, 1.29]	**<0.005**
Ischemic or Hemorrhagic Stroke	1.35 [1.17, 1.57]	**<0.005**	1.10 [0.94, 1.27]	0.23
Major Adverse Cardiovascular Events	1.65 [1.54, 1.77]	**<0.005**	1.13 [1.05, 1.22]	**<0.005**

	B) Inverse Probability Weighting-Adjusted			
Outcome	COVID+ Hospitalized vs COVID–		COVID+ Non-Hospitalized vs COVID–	
	HR [95% CI]	*p*-value	HR [95% CI]	*p*-value
All-Cause Mortality	2.95 [2.51, 3.48]	**<0.005**	1.21 [1.00, 1.47]	0.055
Myocardial Infarction	1.66 [1.38, 1.98]	**<0.005**	1.10 [0.94, 1.30]	0.25
Heart Failure	1.84 [1.64, 2.06]	**<0.005**	1.17 [1.06, 1.30]	**<0.005**
Ischemic or Hemorrhagic Stroke	1.44 [1.20, 1.72]	**<0.005**	1.05 [0.89, 1.23]	0.56
Major Adverse Cardiovascular Events	1.87 [1.72, 2.04]	**<0.005**	1.14 [1.05, 1.23]	**<0.005**

**A)** Multivariate regression and **B)** inverse probability weighting both adjusted for baseline age, sex, race, ethnicity, comorbidities, stage of hypertension, insurance status, tertile of Zone Improvement Plan code median income, presence of unmet social needs, and SARS-CoV-2 vaccination status.

*HR*, hazard ratio; *CI*, confidence interval.

Bold cases indicate statistical significance.

**Fig. 2:**
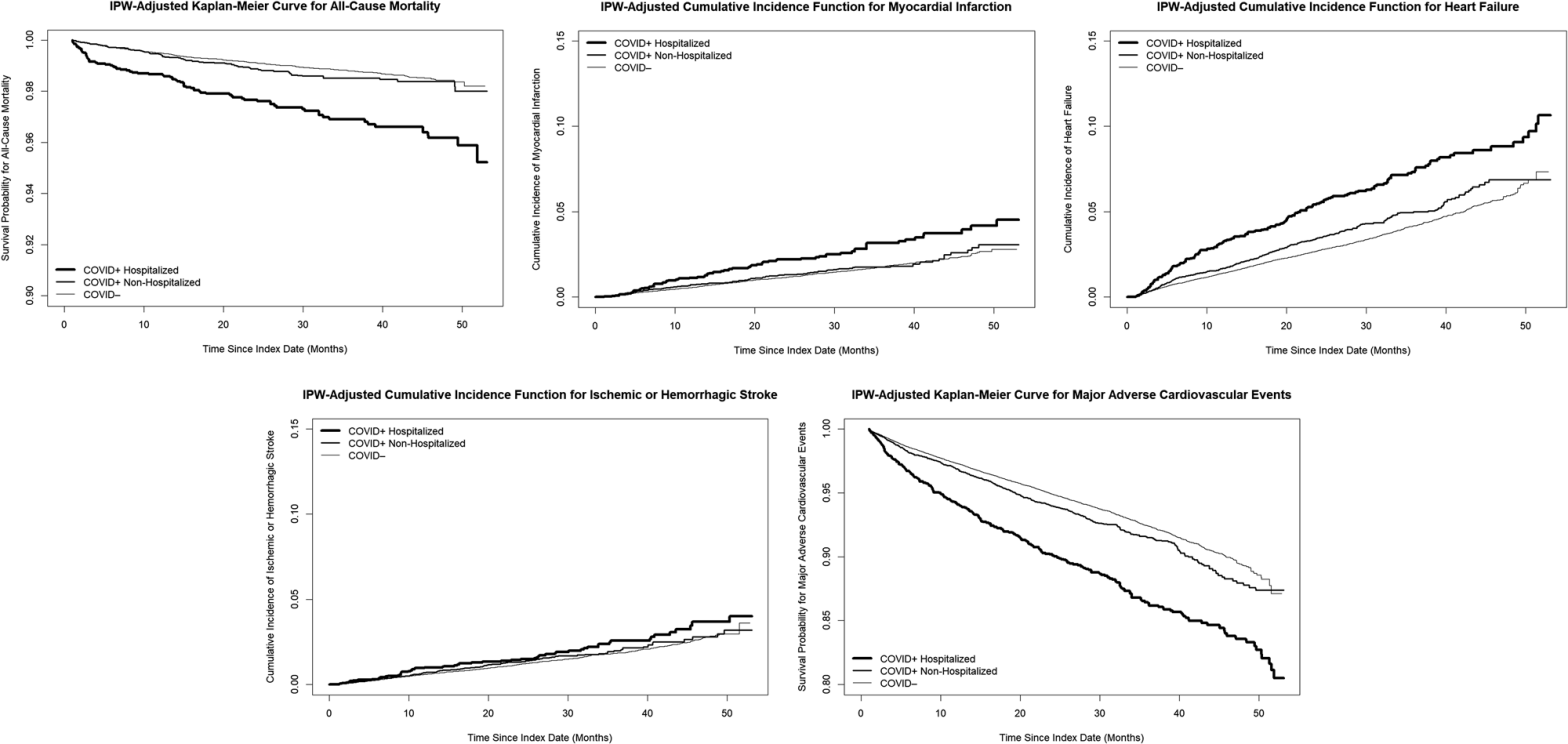
Kaplan-Meier (all-cause mortality and major adverse cardiovascular events) and cumulative incidence function (myocardial infarction, heart failure, and ischemic or hemorrhagic stroke) up to 52 months follow-up among COVID+ hospitalized, COVID+ non-hospitalized, and COVID– groups. The three groups were inverse probability weighting (IPW)-adjusted according to age, sex, race, ethnicity, comorbidities, stage of hypertension, insurance status, tertile of Zone Improvement Plan code median income, presence of unmet social needs, and SARS-CoV-2 vaccination status. IPW, inverse probability weighting.

Analysis was also performed using IPW (Table 2B). Supplementary Table 2 shows the demographic profile of patients before and after IPW was applied. The results are shown as IPW-adjusted Kaplan-Meier and cumulative incidence curves in Fig. 2. The results using IPW were similar to those with multivariate regression.

**Fig. 3:**
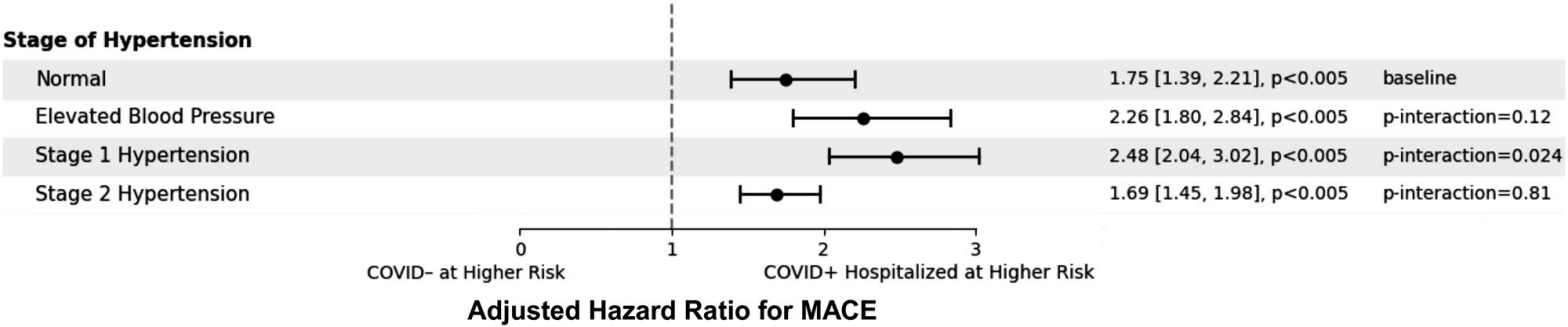
Stratified analysis of the association between COVID-19 hospitalization and risk of major adverse cardiovascular events (MACE), by stage of hypertension. Inverse probability weighting (IPW)-adjusted hazard ratios (HRs) and 95% confidence intervals compare COVID+ hospitalized patients to COVID– individuals within each blood pressure subgroup. Interaction *p*-values reflect whether the effect of COVID-19 hospitalization on MACE differs significantly across hypertension stages. The reference group is the elevated category (baseline). IPW adjusted for baseline age, sex, race, ethnicity, comorbidities, stage of hypertension, insurance status, tertile of Zone Improvement Plan code median income, presence of unmet social needs, and SARS-CoV-2 vaccination status. MACE, major adverse cardiovascular events.

Figure 3 shows IPW-adjusted hazard ratios stratified by baseline hypertension stage, illustrating COVID-19-related risk for MACE differs across BP categories. Interaction terms were used to test whether these subgroup differences were statistically significant. Among individuals with normal BP, hospitalization for COVID-19 was associated with a 1.75-fold increased risk of MACE (95% confidence interval: 1.39, 2021). In contrast, the relative risk associated with COVID-19 hospitalization was higher among individuals with elevated BP (2.26 [1.80, 2.84]) and stage 1 hypertension (2.48 [2.04, 3.02]). Formal interaction testing yielded statistically significant results (*p*-interaction < 0.005 for stage 1 vs normal BP), indicating that the strength of the association between COVID-19 and MACE differed by hypertension stage. A HR of 1.69 [1.45, 1.98] was found in those with stage 2 hypertension. A similar interaction with hypertension stage was not observed for the non-hospitalized COVID-19 cohort (Supplementary Table 3).

To investigate the potential association of outcomes with biomarkers, we evaluated MACE with respect to biomarkers obtained during acute COVID-19. Table 3 shows the IPW-adjusted HRs for biomarkers at time of infection and risk of MACE among patients hospitalized for COVID-19. CRP ≥ 1.1 mg/dL, creatinine ≥ 1.1 mg/dL, LDH ≥ 400 U/L, D-dimer ≥ 1.5 µg/mL, hemoglobin ≤ 9.2 g/dL, and NLR ≥ 10 were associated with increased risk of MACE.

**Table 3:** Cox proportional hazard ratios (HR) for biomarkers at time of infection and risk of major adverse cardiovascular events among patients hospitalized for COVID-19.

Biomarker Predictor	IPW-Adjusted HR [95% CI]	*p*-value
Aspartate Aminotransferase ≥ 100 U/L	1.15 [0.95, 1.40]	0.15
C-Reactive Protein ≥ 15 mg/dL	1.20 [1.04, 1.39]	**0.013**
Creatinine ≥ 1.1 mg/dL	1.38 [1.18, 1.61]	**<0.005**
Lactate Dehydrogenase ≥ 400 U/L	1.16 [1.00, 1.35]	**0.049**
Ferritin ≥ 700 µg/L	1.11 [0.95, 1.29]	0.18
D-dimer ≥ 1.5 µg/mL	1.36 [1.18, 1.55]	**<0.005**
Hemoglobin ≤ 9.2 g/dL	1.89 [1.66, 2.15]	**<0.005**
Neutrophil/Lymphocyte Ratio ≥ 10	1.25 [1.11, 1.41]	**<0.005**
Temperature ≥ 38.0 °C	0.98 [0.87, 1.11]	0.78
Platelets ≤ 110 × 10^9^ cells/L	1.60 [0.96, 2.67]	0.073

Inverse probability weighting (IPW) adjusted for baseline age, sex, race, ethnicity, comorbidities, stage of hypertension, insurance status, tertile of Zone Improvement Plan code median income, presence of unmet social needs, and SARS-CoV-2 vaccination status.

*IPW*, inverse probability weighting; *HR*, hazard ratio; *CI*, confidence interval.

Bold cases indicate statistical significance.

### Sensitivity analyses

Our main analysis excluded patients who were lost to follow-up or experienced MACE within 30 days of the index date, which might have introduced survivor biases. A sensitivity analysis was performed in which these exclusions were not applied (Appendix 2). The results were directionally consistent, though patients with COVID-19 were at much greater risk of mortality if those who died acutely were not excluded.

As there were three definitions for hypertension (based on BP measurements, ICD-10 diagnosis, or antihypertensive use), the association between COVID-19 and MACE within each of the three definitions was investigated. The HRs for MACE to be similar for different definitions used (Supplementary Table 4).

**Fig. 4:**
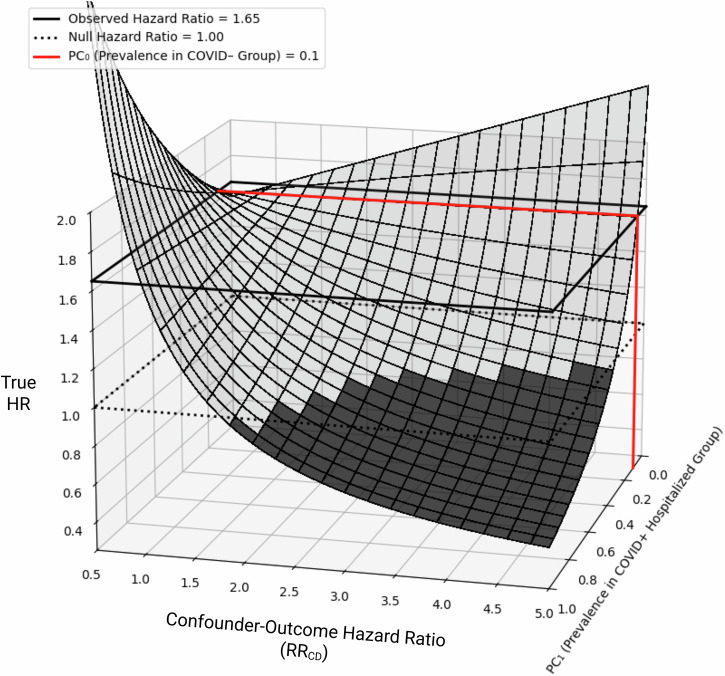
Sensitivity analysis evaluating the potential influence of residual confounding. The surface depicts the true hazard ratio, assuming an observed hazard ratio of 1.65 and a prevalence of the unmeasured confounder (PC_0_) of 0.1 (10%) in the COVID− group. RR_CD_ represents the independent association between an unmeasured confounder and the risk of first-time major adverse cardiovascular events (MACE). If a confounder had an independent risk ratio of 1.75 for new-onset MACE and was at least 3.5 times more prevalent in the COVID+ hospitalized group compared to the COVID − , then the observed effect could be nullified, which is unlikely.

A sensitivity analysis was conducted to evaluate the potential impact of residual confounding on our primary outcome [51]. To fully explain the observed multivariate-adjusted hazard ratio of 1.65 (COVID+ hospitalized for MACE), an unmeasured confounder independently associated with MACE by a HR of 1.75 would need to be at least 3.5 times as prevalent in the COVID+ hospitalized group compared to the COVID− group (Fig. 4).

## Discussion

This is the first study to evaluate the long-term cardiovascular outcomes of COVID-19 survivors with pre-existing hypertension. Over a follow-up period of up to 4.5 years post infection, we found that: **1)** COVID+ hospitalized patients are at higher risk of first-time MI, HF, stroke, all-cause mortality, and MACE compared to COVID− controls after adjusting for demographic, clinical, and socioeconomic variables, as well as SARS-CoV-2 vaccination status; **2)** COVID+ non-hospitalized patients are at higher adjusted risk of HF and MACE compared to COVID− controls; **3)** Risk of MACE was higher in those who were Black, Hispanic, had comorbidities, had at least one unmet social need, or were on Medicare or Medicaid; **4)** The association between COVID+ hospitalization and first-time MACE was amplified in those with stage 1 hypertension compared to those with normal BP; **5)** Among hospitalized COVID+ patients, blood biomarkers at the time of infection (CRP ≥ 1.1 mg/dL, creatinine ≥ 1.1 mg/dL, LDH ≥ 400 U/L, D-dimer ≥ 1.5 µg/mL, hemoglobin ≤ 9.2 g/dL, and NLR ≥ 10) were predictive of future first-time MACE risk. Sub-analyses further confirmed the main findings.

Several prior studies have reported increased risk of first-time cardiovascular disorders, including MACE [13–16, 52–54], hypertension [12], MI [16, 55], HF [15], and stroke [14] among COVID-19 survivors compared to COVID-19 negative controls. However, no studies to our knowledge have specifically evaluated long-term post-COVID cardiovascular outcomes among individuals with pre-existing hypertension. Patients with hypertension are vulnerable to post-COVID cardiovascular complications, likely due to elevated baseline risk profiles and unique pathophysiological features that may be worsen by COVID-19 illness [8]. SARS-CoV-2 virions bind to ACE2 receptors, and that such action leads to buildup of angiotensin II, resulting in vasoconstriction and potential exacerbation of high BP [56, 57]. Hypertensive patients at baseline have a dysregulated renin-angiotensin-aldosterone system (RAAS) [58–60], which may make these individuals more vulnerable to further exacerbation by SARS-CoV-2 infection. Injury to endothelial cells and microvascular structures further promotes thrombogenesis and vascular dysfunction [17, 18]. Moreover, pulmonary stress as the results of COVID-19 illness could further contribute to worsening of hypertension-related complications in patients with pre-existing hypertension [61, 62]. Together, these processes could contribute to worse long-term clinical outcomes in hypertensive individuals with COVID-19 compared to those without COVID-19.

Hypertensive patients hospitalized for COVID-19 had higher elevated risk of outcomes compared to hypertensive patients not hospitalized for COVID-19, suggesting that COVID-19 disease severity contributed to outcomes. This is further supported by analysis of biomarkers within hospitalized COVID-19 patients, which showed that biomarker indicators of more severe infection and inflammatory response (abnormal CRP, creatinine, LDH, D-dimer, hemoglobin, and NLR) at time of infection are predictive of future MACE risk. COVID-19 may also induce systemic inflammation, endothelial dysfunction, hypoxia, and coagulopathy, which in the context of hypertension can exacerbate already elevated thrombotic and cardiovascular risk [17, 18].

Hypertensive patients with non-hospitalized COVID-19 illness were also at elevated risk for outcomes (HF and MACE) compared to COVID− controls. These findings suggest that even mild or asymptomatic SARS-CoV-2 infections may carry lasting cardiovascular consequences. Emerging research has similarly shown that post-acute sequelae of COVID-19 are not confined to those who experienced severe illness requiring hospitalization; rather, subclinical inflammation, endothelial dysfunction, and autonomic dysregulation have been reported in individuals with mild infections, potentially predisposing them to cardiovascular disease in the long term [52, 63, 64]. Clinically, this underscores the importance of long-term cardiovascular monitoring not only in high-risk or hospitalized patients but also in those with milder COVID-19 presentations. Our findings are in general agreement with a previous report that found increased risk of clinical outcomes in COVID-19 survivors with pre-existing CAD [23]. Public health strategies should thus account for this broader at-risk population when designing follow-up care and preventive interventions.

Notably, among hypertensive patients with normal (controlled) BP, hospitalized COVID-19 confers 75% increased risk for MACE, while in those with elevated BP and stage 1 hypertension, hospitalized COVID-19 confers 126% and 148% increased risk, respectively. This finding suggested patients with controlled BP with medication or lifestyle changes in hypertensive patients had reduced risk. However, this trend did not follow in those with stage 2 hypertension, who experienced 69% increased risk of MACE as a result of hospitalized COVID-19. This may be due to a ceiling effect, wherein individuals with stage 2 hypertension already have a substantially elevated baseline cardiovascular risk, such that the relative impact of an acute insult like COVID-19 is proportionally smaller [65].

Risk of MACE was higher in individuals who were on Medicare, Medicaid, or had at least one unmet social need compared to their counterparts. This is consistent with the literature which suggests that low income and limited access to healthcare are linked to higher rates of uncontrolled blood pressure and therefore increased cardiovascular risk [66, 67]. Unmet social needs, which often occur in the context of economic instability, lack of access to education, healthcare and quality, social and community all amplify risk [68, 69]. Data here showed that vaccination for SARS-CoV-2 prior to index date is protective for all-cause mortality, suggesting that in those with baseline hypertension, vaccination may reduce the severity of acute infection and therefore reduce subsequent risk of adverse outcomes [70].

Recognizing the inherent limitations of retrospective designs, we performed quantitative bias analysis to estimate the magnitude of unmeasured confounding required to fully account for the observed association between COVID-19 hospitalization and MACE [51]. Specifically, we found that to attenuate the hazard ratio for MACE in the COVID+ hospitalized group to the null, an unmeasured confounder with a risk ratio of 1.75 for new-onset MACE would need to be at least 3.5-fold more prevalent in the COVID+ hospitalized group than in the COVID– group, an imbalance that is unlikely in practice.

### Limitations

This study has several limitations. We relied on the accuracy of the EHR. Antibody tests and at-home COVID-19 tests were not used because they were less reliable and/or not well-documented. Patients could be misclassified as COVID + , if they were tested positive elsewhere and such misclassification likely underestimated any potential impact of the infection on outcomes. However, cases of severe COVID-19 were less likely to have been missed due to the need for inpatient admission, as Montefiore Health System is the predominant healthcare provider in the Bronx. Associations of various acute COVID-19 treatments with outcomes were not studied because there were many combinations of treatments which were not systematically administered across the pandemic, especially during the early pandemic. The contribution of acute biomarkers to outcomes in the non-hospitalized COVID-19 group was not studied because these laboratory tests were usually not clinically indicated in these patients. As our cohort is diverse, consisting of large proportions of racial minorities in an underserved population, our findings may not be representative of less diverse populations. There could be potential for selection bias introduced by excluding patients who experienced outcomes or were lost to follow-up within 30 days of index date. While this was done to minimize acute-phase confounding and clarify long-term risk, it may have excluded patients at highest baseline risk, thereby underestimating the true association between COVID-19 and MACE. Our sensitivity analysis without this exclusion criterion showed similar results.

Major confounders IPW were accounted for using multivariate regression. The possibility of unmeasured residual confounding was also assessed, and only an extreme scenario of residual confounding could fully explain the observed effect size. Despite our best efforts, unintentional patient selection biases, and other unmeasured biases cannot be entirely excluded from any observational analysis.

## Conclusion

Survivors of hospitalized COVID-19 with pre-existing hypertension are at higher adjusted risk of future first-time MI, HF, stroke, all-cause mortality, and MACE as compared to COVID− patients with hypertension up to 52 months of follow-up. Risk amplification by COVID-19 is more pronounced among those with stage 1 hypertension as compared to those with normal BP. Abnormal levels of CRP, creatinine, LDH, D-dimer, hemoglobin, and NLR during COVID-19 hospitalization are predictive of higher future risk of MACE. These findings underscore the importance of long-term cardiovascular monitoring in this vulnerable population of hypertensive COVID-19 survivors.

## Supplementary information

Supplementary Table 1

Supplementary Table 2

Supplementary Table 3

Supplementary Table 4

Appendix 1

Appendix 2

## Data Availability

The data underlying this study are not publicly available due to institutional and IRB restrictions involving patient privacy and confidentiality. De-identified summary data and analysis code are available from the corresponding author upon reasonable request and contingent upon institutional approval and execution of a data use agreement. Please contact Tim Q. Duong at tim.duong@einsteinmed.edu.
