# Supplementary material for: COVID-19 and cardiovascular outcomes in patients with pre-existing hypertension
Source: J Hum Hypertens. 2026 Apr 9;40(6):446–55. doi: 10.1038/s41371-026-01147-4 (PMC13249568; doi:10.1038/s41371-026-01147-4)
Supplement: Supplementary file 1 — Supplementary Table 1 [file 41371_2026_1147_MOESM1_ESM.docx]

**Supplementary Table 1.** Cox-proportional (all-cause mortality and major adverse cardiovascular events) and Fine-Gray subdistribution (myocardial infarction, congestive heart failure, and ischemic or hemorrhagic stroke) adjusted hazard ratios (HR) for different outcomes grouped by COVID-19 status (COVID+ hospitalized and COVID+ non-hospitalized versus COVID– controls). Multivariate models were adjusted for baseline age, sex, race, ethnicity, comorbidities, stage of hypertension, insurance status, tertile of Zone Improvement Plan median income, presence of unmet social needs, and SARS-CoV-2 vaccination status. COPD, chronic obstructive pulmonary disease. HR, hazard ratio. CI, confidence interval.

| **All-Cause Mortality** | | | | |
| --- | --- | --- | --- | --- |
| **Covariate** | Unadjusted HR [95% CI] | *p*-value | Adjusted HR [95% CI] | *p*-value |
| **COVID-19 Status** |  |  |  |  |
| COVID+ Hospitalized vs COVID– | 5.14 [4.51, 5.85] | **<0.005** | 2.51 [2.17, 2.90] | **<0.005** |
| COVID+ Non-Hospitalized vs COVID– | 0.68 [0.57, 0.82] | **<0.005** | 1.16 [0.96, 1.39] | 0.12 |
| Vaccination for SARS-CoV-2 | 0.74 [0.63, 0.85] | **<0.005** | 0.85 [0.73, 0.99] | **0.037** |
| **Age and Sex** |  |  |  |  |
| Age at Index Date (Years) | 1.07 [1.07, 1.08] | **<0.005** | 1.06 [1.05, 1.06] | **<0.005** |
| Male vs Female | 1.72 [1.52, 1.94] | **<0.005** | 1.44 [1.27, 1.63] | **<0.005** |
| **Race and Ethnicity** |  |  |  |  |
| Black vs Non-Hispanic White | 1.11 [0.98, 1.26] | 0.11 | 1.00 [0.83, 1.21] | 0.99 |
| Asian vs Non-Hispanic White | 0.51 [0.34, 0.77] | **<0.005** | 0.52 [0.33, 0.81] | **<0.005** |
| Other Race vs Non-Hispanic White | 0.77 [0.68, 0.87] | **<0.005** | 0.75 [0.59, 0.96] | **0.020** |
| Hispanic vs Non-Hispanic | 0.79 [0.70, 0.90] | **<0.005** | 1.00 [0.82, 1.22] | 0.98 |
| **Pre-Existing Comorbidities** |  |  |  |  |
| Coronary Artery Disease | 3.18 [2.73, 3.71] | **<0.005** | 1.22 [1.04, 1.44] | **0.014** |
| Type-2 Diabetes | 1.99 [1.76, 2.24] | **<0.005** | 1.10 [0.97, 1.25] | 0.14 |
| COPD | 5.21 [4.37, 6.21] | **<0.005** | 2.13 [1.76, 2.58] | **<0.005** |
| Asthma | 0.86 [0.73, 1.01] | 0.062 | 0.92 [0.78, 1.09] | 0.36 |
| Chronic Kidney Disease | 3.91 [3.44, 4.44] | **<0.005** | 1.75 [1.52, 2.02] | **<0.005** |
| Liver Disease | 2.81 [2.42, 3.26] | **<0.005** | 2.16 [1.86, 2.52] | **<0.005** |
| Tobacco Use | 1.76 [1.56, 1.98] | **<0.005** | 1.23 [1.08, 1.40] | **<0.005** |
| Obesity | 0.68 [0.60, 0.77] | **<0.005** | 0.89 [0.78, 1.01] | 0.065 |
| **Stage of Hypertension** |  |  |  |  |
| Stage 1 Hypertension vs Elevated | 0.55 [0.48, 0.64] | **<0.005** | 0.59 [0.48, 0.74] | **<0.005** |
| Stage 2 Hypertension vs Elevated | 1.06 [0.93, 1.22] | 0.37 | 0.64 [0.52, 0.79] | **<0.005** |
| Elevated vs Normal | 0.88 [0.75, 1.05] | 0.15 | 0.77 [0.61, 0.97] | **0.028** |
| No Blood Pressure Measurements vs Normal | 1.55 [1.35, 1.78] | **<0.005** | 0.79 [0.64, 0.98] | **0.032** |
| **Insurance** |  |  |  |  |
| Medicaid vs Private Insurance | 0.66 [0.58, 0.76] | **<0.005** | 1.70 [1.43, 2.03] | **<0.005** |
| Medicare vs Private Insurance | 3.72 [3.29, 4.20] | **<0.005** | 1.41 [1.20, 1.65] | **<0.005** |
| Uninsured vs Private Insurance | 0.39 [0.27, 0.58] | **<0.005** | 0.95 [0.63, 1.42] | 0.80 |
| **Income Tertile** |  |  |  |  |
| Lower Third vs Top Third | 0.92 [0.81, 1.04] | 0.19 | 0.91 [0.78, 1.05] | 0.21 |
| Middle Third vs Top Third | 0.93 [0.81, 1.07] | 0.30 | 0.85 [0.73, 1.00] | **0.047** |
| **Unmet Social Needs** |  |  |  |  |
| At Least One Unmet Social Need vs None | 0.46 [0.34, 0.62] | **<0.005** | 0.68 [0.50, 0.94] | **0.020** |
| Unmet Social Needs Status Unknown vs None | 1.85 [1.60, 2.13] | **<0.005** | 1.70 [1.45, 1.99] | **<0.005** |

| **Myocardial Infarction** | | | | |
| --- | --- | --- | --- | --- |
| **Covariate** | Unadjusted HR [95% CI] | *p*-value | Adjusted HR [95% CI] | *p*-value |
| **COVID-19 Status** |  |  |  |  |
| COVID+ Hospitalized vs COVID– | 2.54 [2.22, 2.91] | **<0.005** | 1.40 [1.21, 1.63] | **<0.005** |
| COVID+ Non-Hospitalized vs COVID– | 0.81 [0.70, 0.94] | **0.0070** | 1.08 [0.92, 1.26] | 0.34 |
| Vaccination for SARS-CoV-2 | 0.87 [0.76, 1.00] | **0.046** | 0.95 [0.83, 1.09] | 0.47 |
| **Age and Sex** |  |  |  |  |
| Age at Index Date (Years) | 1.05 [1.04, 1.05] | **<0.005** | 1.03 [1.03, 1.04] | **<0.005** |
| Male vs Female | 1.66 [1.49, 1.85] | **<0.005** | 1.45 [1.29, 1.62] | **<0.005** |
| **Race and Ethnicity** |  |  |  |  |
| Black vs Non-Hispanic White | 0.95 [0.85, 1.07] | 0.42 | 1.26 [1.03, 1.54] | **0.026** |
| Asian vs Non-Hispanic White | 0.82 [0.61, 1.10] | 0.18 | 1.00 [0.71, 1.41] | 0.99 |
| Other Race vs Non-Hispanic White | 1.06 [0.95, 1.18] | 0.28 | 1.18 [0.94, 1.49] | 0.16 |
| Hispanic vs Non-Hispanic | 1.05 [0.94, 1.17] | 0.39 | 1.04 [0.88, 1.23] | 0.67 |
| **Pre-Existing Comorbidities** |  |  |  |  |
| Coronary Artery Disease | 4.07 [3.59, 4.63] | **<0.005** | 2.25 [1.96, 2.58] | **<0.005** |
| Type-2 Diabetes | 2.28 [2.05, 2.54] | **<0.005** | 1.38 [1.23, 1.55] | **<0.005** |
| COPD | 2.35 [1.89, 2.91] | **<0.005** | 1.11 [0.89, 1.39] | 0.36 |
| Asthma | 0.97 [0.84, 1.11] | 0.61 | 1.06 [0.92, 1.22] | 0.42 |
| Chronic Kidney Disease | 2.82 [2.50, 3.18] | **<0.005** | 1.55 [1.36, 1.77] | **<0.005** |
| Liver Disease | 1.23 [1.03, 1.46] | **0.023** | 0.96 [0.80, 1.15] | 0.66 |
| Tobacco Use | 1.70 [1.52, 1.89] | **<0.005** | 1.38 [1.23, 1.54] | **<0.005** |
| Obesity | 0.80 [0.72, 0.89] | **<0.005** | 0.91 [0.81, 1.02] | 0.092 |
| **Stage of Hypertension** |  |  |  |  |
| Stage 1 Hypertension vs Elevated | 0.57 [0.50, 0.65] | **<0.005** | 0.83 [0.67, 1.03] | 0.093 |
| Stage 2 Hypertension vs Elevated | 1.25 [1.11, 1.41] | **<0.005** | 1.11 [0.90, 1.36] | 0.35 |
| Elevated vs Normal | 0.62 [0.52, 0.74] | **<0.005** | 0.80 [0.63, 1.03] | 0.080 |
| No Blood Pressure Measurements vs Normal | 1.98 [1.76, 2.22] | **<0.005** | 1.65 [1.33, 2.05] | **<0.005** |
| **Insurance** |  |  |  |  |
| Medicaid vs Private Insurance | 0.84 [0.75, 0.94] | **<0.005** | 1.54 [1.33, 1.78] | **<0.005** |
| Medicare vs Private Insurance | 2.62 [2.34, 2.93] | **<0.005** | 1.35 [1.16, 1.56] | **<0.005** |
| Uninsured vs Private Insurance | 0.56 [0.42, 0.75] | **<0.005** | 1.16 [0.85, 1.57] | 0.34 |
| **Income Tertile** |  |  |  |  |
| Lower Third vs Top Third | 1.01 [0.90, 1.12] | 0.91 | 0.94 [0.82, 1.08] | 0.39 |
| Middle Third vs Top Third | 1.00 [0.89, 1.13] | 0.96 | 0.96 [0.83, 1.10] | 0.56 |
| **Unmet Social Needs** |  |  |  |  |
| At Least One Unmet Social Need vs None | 1.05 [0.87, 1.25] | 0.63 | 1.16 [0.95, 1.43] | 0.15 |
| Unmet Social Needs Status Unknown vs None | 1.11 [0.99, 1.24] | 0.079 | 1.04 [0.91, 1.18] | 0.60 |

| **Heart Failure** | | | | |
| --- | --- | --- | --- | --- |
| **Covariate** | Unadjusted HR [95% CI] | *p*-value | Adjusted HR [95% CI] | *p*-value |
| **COVID-19 Status** |  |  |  |  |
| COVID+ Hospitalized vs COVID– | 2.85 [2.62, 3.11] | **<0.005** | 1.59 [1.45, 1.75] | **<0.005** |
| COVID+ Non-Hospitalized vs COVID– | 0.87 [0.79, 0.96] | **<0.005** | 1.17 [1.06, 1.29] | **<0.005** |
| Vaccination for SARS-CoV-2 | 0.96 [0.88, 1.04] | 0.33 | 1.00 [0.92, 1.09] | 0.95 |
| **Age and Sex** |  |  |  |  |
| Age at Index Date (Years) | 1.05 [1.05, 1.06] | **<0.005** | 1.04 [1.03, 1.04] | **<0.005** |
| Male vs Female | 1.36 [1.27, 1.46] | **<0.005** | 1.28 [1.19, 1.38] | **<0.005** |
| **Race and Ethnicity** |  |  |  |  |
| Black vs Non-Hispanic White | 1.10 [1.02, 1.18] | **0.0090** | 1.12 [0.99, 1.27] | 0.069 |
| Asian vs Non-Hispanic White | 0.72 [0.59, 0.88] | **<0.005** | 0.85 [0.67, 1.06] | 0.15 |
| Other Race vs Non-Hispanic White | 0.90 [0.84, 0.97] | **<0.005** | 0.87 [0.75, 1.01] | 0.065 |
| Hispanic vs Non-Hispanic | 0.97 [0.90, 1.04] | 0.42 | 1.11 [0.99, 1.24] | 0.080 |
| **Pre-Existing Comorbidities** |  |  |  |  |
| Coronary Artery Disease | 3.26 [2.99, 3.57] | **<0.005** | 1.63 [1.48, 1.79] | **<0.005** |
| Type-2 Diabetes | 2.31 [2.15, 2.48] | **<0.005** | 1.33 [1.23, 1.43] | **<0.005** |
| COPD | 3.07 [2.71, 3.48] | **<0.005** | 1.45 [1.27, 1.66] | **<0.005** |
| Asthma | 1.08 [0.99, 1.17] | 0.089 | 1.07 [0.98, 1.17] | 0.13 |
| Chronic Kidney Disease | 3.28 [3.04, 3.54] | **<0.005** | 1.69 [1.55, 1.84] | **<0.005** |
| Liver Disease | 1.34 [1.20, 1.50] | **<0.005** | 0.99 [0.88, 1.10] | 0.82 |
| Tobacco Use | 1.60 [1.49, 1.72] | **<0.005** | 1.26 [1.17, 1.36] | **<0.005** |
| Obesity | 1.01 [0.94, 1.08] | 0.88 | 1.11 [1.03, 1.20] | **0.0060** |
| **Stage of Hypertension** |  |  |  |  |
| Stage 1 Hypertension vs Elevated | 0.62 [0.57, 0.67] | **<0.005** | 0.73 [0.64, 0.83] | **<0.005** |
| Stage 2 Hypertension vs Elevated | 1.37 [1.27, 1.47] | **<0.005** | 0.95 [0.84, 1.08] | 0.46 |
| Elevated vs Normal | 0.58 [0.52, 0.65] | **<0.005** | 0.63 [0.54, 0.73] | **<0.005** |
| No Blood Pressure Measurements vs Normal | 1.58 [1.46, 1.71] | **<0.005** | 1.22 [1.07, 1.39] | **<0.005** |
| **Insurance** |  |  |  |  |
| Medicaid vs Private Insurance | 0.71 [0.66, 0.77] | **<0.005** | 1.39 [1.27, 1.54] | **<0.005** |
| Medicare vs Private Insurance | 2.98 [2.77, 3.20] | **<0.005** | 1.44 [1.31, 1.59] | **<0.005** |
| Uninsured vs Private Insurance | 0.47 [0.39, 0.58] | **<0.005** | 0.99 [0.81, 1.23] | 0.96 |
| **Income Tertile** |  |  |  |  |
| Lower Third vs Top Third | 1.07 [1.00, 1.15] | 0.057 | 1.02 [0.94, 1.12] | 0.63 |
| Middle Third vs Top Third | 0.97 [0.89, 1.04] | 0.38 | 0.95 [0.87, 1.04] | 0.28 |
| **Unmet Social Needs** |  |  |  |  |
| At Least One Unmet Social Need vs None | 1.24 [1.11, 1.39] | **<0.005** | 1.20 [1.06, 1.36] | **<0.005** |
| Unmet Social Needs Status Unknown vs None | 0.91 [0.84, 0.97] | **0.0080** | 0.92 [0.85, 1.00] | 0.054 |

| **Ischemic or Hemorrhagic Stroke** | | | | |
| --- | --- | --- | --- | --- |
| **Covariate** | Unadjusted HR [95% CI] | *p*-value | Adjusted HR [95% CI] | *p*-value |
| **COVID-19 Status** |  |  |  |  |
| COVID+ Hospitalized vs COVID– | 2.31 [2.00, 2.65] | **<0.005** | 1.35 [1.17, 1.57] | **<0.005** |
| COVID+ Non-Hospitalized vs COVID– | 0.86 [0.74, 1.00] | **0.043** | 1.10 [0.94, 1.27] | 0.23 |
| Vaccination for SARS-CoV-2 | 0.94 [0.82, 1.06] | 0.31 | 0.96 [0.85, 1.10] | 0.59 |
| **Age and Sex** |  |  |  |  |
| Age at Index Date (Years) | 1.05 [1.04, 1.05] | **<0.005** | 1.04 [1.03, 1.04] | **<0.005** |
| Male vs Female | 1.13 [1.01, 1.26] | **0.031** | 1.09 [0.97, 1.22] | 0.14 |
| **Race and Ethnicity** |  |  |  |  |
| Black vs Non-Hispanic White | 1.12 [1.00, 1.25] | **0.048** | 1.27 [1.04, 1.55] | **0.019** |
| Asian vs Non-Hispanic White | 0.96 [0.73, 1.26] | 0.75 | 1.16 [0.84, 1.60] | 0.38 |
| Other Race vs Non-Hispanic White | 0.90 [0.81, 1.01] | 0.067 | 0.93 [0.73, 1.18] | 0.56 |
| Hispanic vs Non-Hispanic | 1.00 [0.90, 1.12] | 0.98 | 1.20 [1.01, 1.43] | **0.043** |
| **Pre-Existing Comorbidities** |  |  |  |  |
| Coronary Artery Disease | 2.20 [1.89, 2.57] | **<0.005** | 1.18 [1.00, 1.38] | **0.046** |
| Type-2 Diabetes | 2.23 [2.01, 2.48] | **<0.005** | 1.44 [1.29, 1.62] | **<0.005** |
| COPD | 2.44 [1.98, 3.00] | **<0.005** | 1.33 [1.06, 1.65] | **0.013** |
| Asthma | 1.16 [1.02, 1.31] | **0.027** | 1.20 [1.05, 1.37] | **0.0090** |
| Chronic Kidney Disease | 2.57 [2.27, 2.90] | **<0.005** | 1.42 [1.24, 1.62] | **<0.005** |
| Liver Disease | 1.23 [1.04, 1.47] | **0.018** | 0.99 [0.83, 1.18] | 0.88 |
| Tobacco Use | 1.37 [1.23, 1.52] | **<0.005** | 1.16 [1.04, 1.30] | **0.009** |
| Obesity | 0.85 [0.76, 0.95] | **<0.005** | 0.92 [0.82, 1.03] | 0.13 |
| **Stage of Hypertension** |  |  |  |  |
| Stage 1 Hypertension vs Elevated | 0.55 [0.48, 0.63] | **<0.005** | 0.69 [0.56, 0.85] | **<0.005** |
| Stage 2 Hypertension vs Elevated | 1.29 [1.14, 1.45] | **<0.005** | 0.96 [0.78, 1.17] | 0.66 |
| Elevated vs Normal | 0.71 [0.60, 0.83] | **<0.005** | 0.77 [0.61, 0.97] | **0.027** |
| No Blood Pressure Measurements vs Normal | 1.72 [1.53, 1.94] | **<0.005** | 1.34 [1.09, 1.64] | **0.0060** |
| **Insurance** |  |  |  |  |
| Medicaid vs Private Insurance | 0.74 [0.66, 0.83] | **<0.005** | 1.33 [1.15, 1.54] | **<0.005** |
| Medicare vs Private Insurance | 2.57 [2.30, 2.87] | **<0.005** | 1.28 [1.11, 1.48] | **<0.005** |
| Uninsured vs Private Insurance | 0.64 [0.49, 0.84] | **<0.005** | 1.20 [0.90, 1.60] | 0.21 |
| **Income Tertile** |  |  |  |  |
| Lower Third vs Top Third | 1.00 [0.90, 1.12] | 0.97 | 0.94 [0.82, 1.08] | 0.38 |
| Middle Third vs Top Third | 0.99 [0.88, 1.12] | 0.93 | 0.94 [0.82, 1.08] | 0.40 |
| **Unmet Social Needs** |  |  |  |  |
| At Least One Unmet Social Need vs None | 1.21 [1.02, 1.43] | **0.030** | 1.15 [0.95, 1.39] | 0.16 |
| Unmet Social Needs Status Unknown vs None | 0.88 [0.79, 0.98] | **0.024** | 0.86 [0.76, 0.98] | **0.021** |

| **Major Adverse Cardiovascular Events** | | | | |
| --- | --- | --- | --- | --- |
| **Covariate** | Unadjusted HR [95% CI] | *p*-value | Adjusted HR [95% CI] | *p*-value |
| **COVID-19 Status** |  |  |  |  |
| COVID+ Hospitalized vs COVID– | 2.89 [2.71, 3.08] | **<0.005** | 1.65 [1.54, 1.77] | **<0.005** |
| COVID+ Non-Hospitalized vs COVID– | 0.84 [0.78, 0.90] | **<0.005** | 1.13 [1.05, 1.22] | **<0.005** |
| Vaccination for SARS-CoV-2 | 0.89 [0.84, 0.95] | **<0.005** | 0.95 [0.89, 1.01] | 0.085 |
| **Age and Sex** |  |  |  |  |
| Age at Index Date (Years) | 1.05 [1.05, 1.05] | **<0.005** | 1.04 [1.04, 1.04] | **<0.005** |
| Male vs Female | 1.39 [1.32, 1.46] | **<0.005** | 1.29 [1.22, 1.37] | **<0.005** |
| **Race and Ethnicity** |  |  |  |  |
| Black vs Non-Hispanic White | 1.08 [1.02, 1.14] | **0.0080** | 1.17 [1.07, 1.28] | **<0.005** |
| Asian vs Non-Hispanic White | 0.76 [0.65, 0.88] | **<0.005** | 0.90 [0.76, 1.06] | 0.21 |
| Other Race vs Non-Hispanic White | 0.92 [0.87, 0.96] | **<0.005** | 0.92 [0.82, 1.03] | 0.15 |
| Hispanic vs Non-Hispanic | 0.97 [0.92, 1.02] | 0.28 | 1.10 [1.01, 1.19] | **0.031** |
| **Pre-Existing Comorbidities** |  |  |  |  |
| Coronary Artery Disease | 3.15 [2.95, 3.37] | **<0.005** | 1.60 [1.49, 1.72] | **<0.005** |
| Type-2 Diabetes | 2.14 [2.04, 2.26] | **<0.005** | 1.28 [1.21, 1.35] | **<0.005** |
| COPD | 3.02 [2.75, 3.33] | **<0.005** | 1.44 [1.30, 1.59] | **<0.005** |
| Asthma | 1.07 [1.00, 1.14] | **0.044** | 1.10 [1.03, 1.18] | **0.0050** |
| Chronic Kidney Disease | 2.94 [2.77, 3.12] | **<0.005** | 1.53 [1.44, 1.63] | **<0.005** |
| Liver Disease | 1.51 [1.39, 1.63] | **<0.005** | 1.14 [1.05, 1.24] | **<0.005** |
| Tobacco Use | 1.56 [1.49, 1.65] | **<0.005** | 1.25 [1.18, 1.32] | **<0.005** |
| Obesity | 0.90 [0.86, 0.95] | **<0.005** | 1.01 [0.96, 1.07] | 0.62 |
| **Stage of Hypertension** |  |  |  |  |
| Stage 1 Hypertension vs Elevated | 0.60 [0.56, 0.64] | **<0.005** | 0.71 [0.64, 0.78] | **<0.005** |
| Stage 2 Hypertension vs Elevated | 1.27 [1.20, 1.35] | **<0.005** | 0.90 [0.82, 0.99] | **0.037** |
| Elevated vs Normal | 0.69 [0.64, 0.74] | **<0.005** | 0.72 [0.64, 0.80] | **<0.005** |
| No Blood Pressure Measurements vs Normal | 1.60 [1.51, 1.70] | **<0.005** | 1.16 [1.05, 1.28] | **<0.005** |
| **Insurance** |  |  |  |  |
| Medicaid vs Private Insurance | 0.74 [0.70, 0.79] | **<0.005** | 1.40 [1.30, 1.50] | **<0.005** |
| Medicare vs Private Insurance | 2.80 [2.66, 2.96] | **<0.005** | 1.34 [1.25, 1.44] | **<0.005** |
| Uninsured vs Private Insurance | 0.53 [0.46, 0.61] | **<0.005** | 1.05 [0.91, 1.22] | 0.49 |
| **Income Tertile** |  |  |  |  |
| Lower Third vs Top Third | 1.03 [0.98, 1.09] | 0.21 | 0.99 [0.93, 1.06] | 0.73 |
| Middle Third vs Top Third | 0.97 [0.92, 1.03] | 0.32 | 0.94 [0.88, 1.01] | 0.074 |
| **Unmet Social Needs** |  |  |  |  |
| At Least One Unmet Social Need vs None | 1.09 [1.00, 1.19] | **0.048** | 1.14 [1.03, 1.26] | **0.0090** |
| Unmet Social Needs Status Unknown vs None | 1.03 [0.97, 1.08] | 0.33 | 1.02 [0.96, 1.09] | 0.52 |
