# Supplementary material for: COVID-19 and cardiovascular outcomes in patients with pre-existing hypertension
Source: J Hum Hypertens. 2026 Apr 9;40(6):446–55. doi: 10.1038/s41371-026-01147-4 (PMC13249568; doi:10.1038/s41371-026-01147-4)
Supplement: Supplementary file 2 — Supplementary Table 2 [file 41371_2026_1147_MOESM2_ESM.docx]

**Supplementary Table 2. A)** Characteristics of cohort before inverse probability weighting and **B)** characteristics of pseudo-population after inverse probability weighting. SD, standard deviation. SMD, standardized mean difference. COPD, chronic obstructive pulmonary disease.

| **A)** | **COVID+ Hospitalized**  **(n=6461)** | **COVID+ Non-Hospitalized**  **(n=15686)** | **COVID−**  **(n=53006)** | **COVID+ Hospitalized vs COVID−** | | **COVID+ Non-Hospitalized vs COVID−** | |
| --- | --- | --- | --- | --- | --- | --- | --- |
|  |  |  |  | ***p*-value** | **SMD** | ***p*-value** | **SMD** |
| Follow Up Time (Months), mean ± SD | 26.75 ± 16.04 | 23.62 ± 12.54 | 27.33 ± 14.27 | **0.0055** | 0.038 | **<0.005** | 0.28 |
| Age at Index Date (Years), mean ± SD | 61.79 ± 17.25 | 52.71 ± 15.79 | 54.68 ± 15.64 | **<0.005** | 0.43 | **<0.005** | 0.13 |
| Female, n (%) | 3723 (57.62%) | 10440 (66.56%) | 33058 (62.37%) | **<0.005** | 0.097 | **<0.005** | 0.088 |
| **Race and Ethnicity, n (%)** |  |  |  |  |  |  |  |
| Non-Hispanic White | 642 (9.94%) | 1411 (9.00%) | 5345 (10.08%) | 0.73 | 0.0049 | **<0.005** | 0.037 |
| Black | 2332 (36.09%) | 5638 (35.94%) | 18331 (34.58%) | **0.017** | 0.032 | **<0.005** | 0.028 |
| Asian | 250 (3.87%) | 775 (4.94%) | 2153 (4.06%) | 0.48 | 0.0099 | **<0.005** | 0.042 |
| Other Race | 3237 (50.10%) | 7862 (50.12%) | 27177 (51.27%) | 0.078 | 0.023 | **0.012** | 0.023 |
| Hispanic | 2783 (43.07%) | 6558 (41.81%) | 21915 (41.34%) | **0.0080** | 0.035 | 0.30 | 0.0094 |
| **Blood Pressure (mm Hg), mean ± SD** |  |  |  |  |  |  |  |
| Systolic Blood Pressure | 133.85 ± 15.23 | 132.74 ± 13.26 | 133.91 ± 14.21 | 0.78 | 0.0045 | **<0.005** | 0.086 |
| Diastolic Blood Pressure | 76.59 ± 8.94 | 79.46 ± 8.12 | 80.02 ± 8.21 | **<0.005** | 0.40 | **<0.005** | 0.069 |
| **Stage of Hypertension, n (%)** |  |  |  |  |  |  |  |
| No Blood Pressure Measurements Available | 1990 (30.80%) | 1975 (12.59%) | 11083 (20.91%) | **<0.005** | 0.23 | **<0.005** | 0.22 |
| Normal | 737 (11.41%) | 1478 (9.42%) | 3838 (7.24%) | **<0.005** | 0.14 | **<0.005** | 0.079 |
| Elevated | 939 (14.53%) | 3005 (19.16%) | 8351 (15.75%) | **0.011** | 0.034 | **<0.005** | 0.09 |
| Stage 1 Hypertension | 1374 (21.27%) | 5406 (34.46%) | 16611 (31.34%) | **<0.005** | 0.23 | **<0.005** | 0.067 |
| Stage 2 Hypertension | 1421 (21.99%) | 3822 (24.37%) | 13123 (24.76%) | **<0.005** | 0.065 | 0.32 | 0.0091 |
| **Definition of Hypertension Met, n (%)** |  |  |  |  |  |  |  |
| Blood Pressure Measurements | 3734 (57.79%) | 12233 (77.99%) | 38085 (71.85%) | **<0.005** | 0.30 | **<0.005** | 0.14 |
| Antihypertensive Use | 4896 (75.78%) | 8465 (53.97%) | 29535 (55.72%) | **<0.005** | 0.43 | **<0.005** | 0.035 |
| ICD-10 Code | 5542 (85.78%) | 9745 (62.13%) | 33288 (62.80%) | **<0.005** | 0.54 | 0.13 | 0.014 |
| **Pre-Existing Comorbidities, n (%)** |  |  |  |  |  |  |  |
| Coronary Artery Disease | 952 (14.73%) | 1140 (7.27%) | 3222 (6.08%) | **<0.005** | 0.29 | **<0.005** | 0.048 |
| Type-2 Diabetes | 3112 (48.17%) | 4400 (28.05%) | 14308 (26.99%) | **<0.005** | 0.45 | **0.0092** | 0.024 |
| COPD | 564 (8.73%) | 429 (2.73%) | 1214 (2.29%) | **<0.005** | 0.29 | **<0.005** | 0.028 |
| Asthma | 1451 (22.46%) | 3650 (23.27%) | 9210 (17.38%) | **<0.005** | 0.13 | **<0.005** | 0.15 |
| Chronic Kidney Disease | 1793 (27.75%) | 1743 (11.11%) | 5106 (9.63%) | **<0.005** | 0.48 | **<0.005** | 0.049 |
| Liver Disease | 766 (11.86%) | 1360 (8.67%) | 4027 (7.60%) | **<0.005** | 0.14 | **<0.005** | 0.039 |
| Obesity | 3758 (58.16%) | 9282 (59.17%) | 28537 (53.84%) | **<0.005** | 0.087 | **<0.005** | 0.11 |
| Tobacco Use | 2461 (38.09%) | 5181 (33.03%) | 18757 (35.39%) | **<0.005** | 0.056 | **<0.005** | 0.050 |
| **Insurance, n (%)** |  |  |  |  |  |  |  |
| Medicaid | 2214 (34.27%) | 5359 (34.16%) | 19259 (36.33%) | **<0.005** | 0.043 | **<0.005** | 0.045 |
| Medicare | 2175 (33.66%) | 2146 (13.68%) | 9249 (17.45%) | **<0.005** | 0.38 | **<0.005** | 0.10 |
| Private | 1873 (28.99%) | 6708 (42.76%) | 21185 (39.97%) | **<0.005** | 0.23 | **<0.005** | 0.057 |
| Uninsured | 199 (3.08%) | 1473 (9.39%) | 3313 (6.25%) | **<0.005** | 0.15 | **<0.005** | 0.12 |
| **Income Group, n (%)** |  |  |  |  |  |  |  |
| Lower Third (≤$42,639/year) | 2750 (42.56%) | 6192 (39.47%) | 21668 (40.88%) | **0.0097** | 0.034 | **<0.005** | 0.029 |
| Middle Third ($42,834/year–$61,272/year) | 1894 (29.31%) | 4642 (29.59%) | 15009 (28.32%) | 0.096 | 0.022 | **<0.005** | 0.028 |
| Top Third (≥$61,414/year) | 1817 (28.12%) | 4852 (30.93%) | 16329 (30.81%) | **<0.005** | 0.059 | 0.77 | 0.0027 |
| **Unmet Social Needs, n (%)** |  |  |  |  |  |  |  |
| At Least One Unmet Social Need | 621 (9.61%) | 1459 (9.30%) | 4258 (8.03%) | **<0.005** | 0.056 | **<0.005** | 0.045 |
| No Unmet Social Needs | 1509 (23.36%) | 4553 (29.03%) | 13405 (25.29%) | **<0.005** | 0.045 | **<0.005** | 0.084 |
| Status Unknown | 4331 (67.03%) | 9674 (61.67%) | 35343 (66.68%) | 0.58 | 0.0076 | **<0.005** | 0.10 |
| **Vaccinated for SARS-CoV-2, n (%)** | 1337 (20.69%) | 7308 (46.59%) | 15940 (30.07%) | **<0.005** | 0.22 | **<0.005** | 0.34 |
| **Outcomes, n (%)** |  |  |  |  |  |  |  |
| All-Cause Mortality | 342 (5.29%) | 146 (0.93%) | 560 (1.06%) | **<0.005** | 0.24 | 0.18 | 0.013 |
| Myocardial Infarction | 260 (4.02%) | 204 (1.30%) | 859 (1.62%) | **<0.005** | 0.15 | **<0.005** | 0.027 |
| Heart Failure | 655 (10.14%) | 523 (3.33%) | 1967 (3.71%) | **<0.005** | 0.26 | **0.028** | 0.020 |
| Ischemic or Hemorrhagic Stroke | 241 (3.73%) | 218 (1.39%) | 882 (1.66%) | **<0.005** | 0.13 | **0.018** | 0.022 |
| Major Adverse Cardiovascular Events | 1175 (18.19%) | 913 (5.82%) | 3544 (6.69%) | **<0.005** | 0.35 | **<0.005** | 0.036 |

| **B)** | **COVID+ Hospitalized**  **(n=6376)** | **COVID+ Non-Hospitalized**  **(n=15845)** | **COVID−**  **(n=52973)** | **COVID+ Hospitalized vs COVID−** | | **COVID+ Non-Hospitalized vs COVID−** | |
| --- | --- | --- | --- | --- | --- | --- | --- |
|  |  |  |  | ***p*-value** | **SMD** | ***p*-value** | **SMD** |
| Follow Up Time (Months), mean ± SD | 25.52 ± 15.60 | 24.29 ± 13.09 | 27.23 ± 14.23 | **0.0055** | 0.11 | **<0.005** | 0.21 |
| Age at Index Date (Years), mean ± SD | 54.01 ± 18.12 | 54.98 ± 16.06 | 54.90 ± 15.78 | **<0.005** | 0.053 | **<0.005** | 0.0048 |
| Female, n (%) | 4179 (65.55%) | 9840 (62.10%) | 33283 (62.83%) | **<0.005** | 0.057 | 0.097 | 0.015 |
| **Race and Ethnicity, n (%)** |  |  |  |  |  |  |  |
| Non-Hispanic White | 583 (9.15%) | 1567 (9.90%) | 5194 (9.81%) | 0.10 | 0.022 | 0.75 | 0.003 |
| Black | 2183 (34.24%) | 5516 (34.82%) | 18544 (35.01%) | 0.23 | 0.016 | 0.67 | 0.004 |
| Asian | 264 (4.15%) | 649 (4.10%) | 2237 (4.22%) | 0.80 | 0.0038 | 0.5 | 0.0064 |
| Other Race | 3345 (52.46%) | 8111 (51.19%) | 26997 (50.96%) | **0.025** | 0.030 | 0.62 | 0.0045 |
| Hispanic | 2830 (44.39%) | 6633 (41.86%) | 22066 (41.66%) | **<0.005** | 0.055 | 0.65 | 0.0042 |
| **Blood Pressure (mm Hg), mean ± SD** |  |  |  |  |  |  |  |
| Systolic Blood Pressure | 135.62 ± 12.99 | 135.65 ± 12.71 | 135.69 ± 13.21 | **<0.005** | 0.0056 | **<0.005** | 0.0028 |
| Diastolic Blood Pressure | 79.46 ± 8.58 | 80.38 ± 8.02 | 80.72 ± 7.95 | **<0.005** | 0.15 | **<0.005** | 0.042 |
| **Stage of Hypertension, n (%)** |  |  |  |  |  |  |  |
| No Blood Pressure Measurements Available | 1181 (18.53%) | 3311 (20.90%) | 10601 (20.01%) | **0.0053** | 0.038 | **0.016** | 0.022 |
| Normal | 720 (11.30%) | 1271 (8.02%) | 4281 (8.08%) | **<0.005** | 0.11 | 0.83 | 0.0021 |
| Elevated | 1129 (17.71%) | 2565 (16.19%) | 8665 (16.36%) | **0.0065** | 0.036 | 0.62 | 0.0046 |
| Stage 1 Hypertension | 1907 (29.91%) | 4856 (30.65%) | 16469 (31.09%) | 0.056 | 0.026 | 0.30 | 0.0096 |
| Stage 2 Hypertension | 1438 (22.55%) | 3840 (24.24%) | 12954 (24.46%) | **<0.005** | 0.045 | 0.59 | 0.005 |
| **Definition of Hypertension Met, n (%)** |  |  |  |  |  |  |  |
| Blood Pressure Measurements | 4474 (70.17%) | 11262 (71.08%) | 38090 (71.90%) | **<0.005** | 0.038 | **0.044** | 0.018 |
| Antihypertensive Use | 4052 (63.55%) | 8845 (55.82%) | 30029 (56.69%) | **<0.005** | 0.14 | 0.055 | 0.017 |
| ICD-10 Code | 4764 (74.72%) | 10545 (66.55%) | 33711 (63.64%) | **<0.005** | 0.24 | **<0.005** | 0.061 |
| **Pre-Existing Comorbidities, n (%)** |  |  |  |  |  |  |  |
| Coronary Artery Disease | 502 (7.88%) | 1124 (7.10%) | 3779 (7.14%) | **0.031** | 0.028 | 0.89 | 0.0014 |
| Type-2 Diabetes | 1928 (30.25%) | 4668 (29.46%) | 15426 (29.12%) | 0.063 | 0.025 | 0.41 | 0.0076 |
| COPD | 204 (3.21%) | 450 (2.84%) | 1574 (2.97%) | 0.32 | 0.013 | 0.41 | 0.0077 |
| Asthma | 1425 (22.36%) | 2972 (18.76%) | 10116 (19.10%) | **<0.005** | 0.080 | 0.34 | 0.0087 |
| Chronic Kidney Disease | 793 (12.44%) | 1823 (11.51%) | 6140 (11.59%) | **0.049** | 0.026 | 0.77 | 0.0027 |
| Liver Disease | 582 (9.13%) | 1269 (8.01%) | 4329 (8.17%) | **0.0092** | 0.034 | 0.52 | 0.006 |
| Obesity | 3717 (58.29%) | 8689 (54.84%) | 29322 (55.35%) | **<0.005** | 0.059 | 0.26 | 0.01 |
| Tobacco Use | 2421 (37.98%) | 5539 (34.96%) | 18632 (35.17%) | **<0.005** | 0.058 | 0.62 | 0.0045 |
| **Insurance, n (%)** |  |  |  |  |  |  |  |
| Medicaid | 2362 (37.05%) | 5729 (36.16%) | 18909 (35.70%) | **0.035** | 0.028 | 0.29 | 0.0097 |
| Medicare | 1178 (18.48%) | 2934 (18.52%) | 9605 (18.13%) | 0.51 | 0.0090 | 0.28 | 0.0099 |
| Private | 2457 (38.54%) | 6207 (39.18%) | 20992 (39.63%) | 0.097 | 0.022 | 0.31 | 0.0092 |
| Uninsured | 378 (5.93%) | 973 (6.14%) | 3466 (6.54%) | 0.064 | 0.025 | 0.075 | 0.016 |
| **Income Group, n (%)** |  |  |  |  |  |  |  |
| Lower Third (≤$42,639/year) | 2691 (42.20%) | 6519 (41.14%) | 21602 (40.78%) | **0.030** | 0.029 | 0.42 | 0.0074 |
| Middle Third ($42,834/year–$61,272/year) | 1816 (28.48%) | 4517 (28.51%) | 15200 (28.69%) | 0.73 | 0.0048 | 0.66 | 0.0041 |
| Top Third (≥$61,414/year) | 1869 (29.32%) | 4808 (30.35%) | 16170 (30.53%) | **0.049** | 0.026 | 0.67 | 0.0039 |
| **Unmet Social Needs, n (%)** |  |  |  |  |  |  |  |
| At Least One Unmet Social Need | 655 (10.27%) | 1321 (8.34%) | 4475 (8.45%) | **<0.005** | 0.063 | 0.68 | 0.0039 |
| No Unmet Social Needs | 1746 (27.38%) | 4076 (25.72%) | 13729 (25.92%) | **0.012** | 0.033 | 0.63 | 0.0044 |
| Status Unknown | 3975 (62.34%) | 10447 (65.94%) | 34768 (65.63%) | **<0.005** | 0.069 | 0.49 | 0.0064 |
| **Vaccinated for SARS-CoV-2, n (%)** | 2110 (33.10%) | 5019 (31.68%) | 17259 (32.58%) | 0.41 | 0.011 | **0.034** | 0.019 |
| **Outcomes, n (%)** |  |  |  |  |  |  |  |
| All-Cause Mortality | 199 (3.12%) | 195 (1.23%) | 591 (1.12%) | **<0.005** | 0.14 | 0.24 | 0.011 |
| Myocardial Infarction | 169 (2.66%) | 261 (1.65%) | 899 (1.70%) | **<0.005** | 0.066 | 0.72 | 0.0036 |
| Heart Failure | 426 (6.69%) | 646 (4.08%) | 2076 (3.92%) | **<0.005** | 0.12 | 0.38 | 0.0081 |
| Ischemic or Hemorrhagic Stroke | 149 (2.34%) | 253 (1.60%) | 917 (1.73%) | **<0.005** | 0.043 | 0.28 | 0.010 |
| Major Adverse Cardiovascular Events | 773 (12.12%) | 1127 (7.11%) | 3703 (6.99%) | **<0.005** | 0.18 | 0.61 | 0.0048 |
