# Supplementary material for: COVID-19 and cardiovascular outcomes in patients with pre-existing hypertension
Source: J Hum Hypertens. 2026 Apr 9;40(6):446–55. doi: 10.1038/s41371-026-01147-4 (PMC13249568; doi:10.1038/s41371-026-01147-4)
Supplement: Supplementary file 3 — Supplementary Table 3 [file 41371_2026_1147_MOESM3_ESM.docx]

**Supplementary Table 3.** Stratified analysis of the association between COVID-19 and risk of major adverse cardiovascular events (MACE), by stage of hypertension. Inverse probability weighting (IPW) adjusted for baseline age, sex, race, ethnicity, comorbidities, insurance status, tertile of Zone Improvement Plan median income, presence of unmet social needs, and SARS-CoV-2 vaccination status. MACE, major adverse cardiovascular events. HR, hazard ratio. CI, confidence interval.

| **Subgroup**  **(Stage of Hypertension)** | HR [95% CI] for MACE | | | |
| --- | --- | --- | --- | --- |
|  | COVID+ Hospitalized vs COVID– | Interaction *p*-value | COVID+ Hospitalized vs COVID– | Interaction *p*-value |
| Normal | 1.75 [1.39, 2.21], *p*<**0.005** | Reference | 1.19 [0.95, 1.49], *p*=0.13 | Reference |
| Elevated Blood Pressure | 2.26 [1.80, 2.84], *p***<0.005** | 0.12 | 1.02 [0.83, 1.25], *p*=0.87 | 0.32 |
| Stage 1 Hypertension | 2.48 [2.04, 3.02], *p***<0.005** | **0.024** | 1.14 [0.97, 1.33], *p*=0.10 | 0.76 |
| Stage 2 Hypertension | 1.70 [1.45, 1.99], *p***<0.005** | 0.81 | 1.18 [1.03, 1.35], *p*=**0.017** | 0.95 |
| No Blood Pressure Measurements | 1.51 [1.30, 1.75], *p*<**0.005** | 0.29 | 1.09 [0.91, 1.29], *p*=0.36 | 0.55 |
