# Supplementary material for: COVID-19 and cardiovascular outcomes in patients with pre-existing hypertension
Source: J Hum Hypertens. 2026 Apr 9;40(6):446–55. doi: 10.1038/s41371-026-01147-4 (PMC13249568; doi:10.1038/s41371-026-01147-4)
Supplement: Supplementary file 4 — Supplementary Table 4 [file 41371_2026_1147_MOESM4_ESM.docx]

**Supplementary Table 4.** Stratified analysis of the association between COVID-19 and risk of major adverse cardiovascular events (MACE), by definition of hypertension met. Inverse probability weighting (IPW) adjusted for baseline age, sex, race, ethnicity, comorbidities, stage of hypertension, insurance status, tertile of Zone Improvement Plan median income, presence of unmet social needs, and SARS-CoV-2 vaccination status. MACE, major adverse cardiovascular events. HR, hazard ratio. CI, confidence interval. ICD-10, International Classification of Diseases, 10th Revision.

| **Subgroup**  **(Definition of Hypertension Met)** | COVID+ Hospitalized vs COVID– | | COVID+ Non-Hospitalized vs COVID– | |
| --- | --- | --- | --- | --- |
|  | MACE HR [95% CI] | *p*-value | MACE HR [95% CI] | *p*-value |
| Blood Pressure | 2.06 [1.84, 2.29] | **<0.005** | 1.13 [1.03, 1.24] | **0.0080** |
| ICD-10 | 1.75 [1.61, 1.91] | **<0.005** | 1.14 [1.05, 1.24] | **<0.005** |
| Antihypertensives | 1.77 [1.62, 1.93] | **<0.005** | 1.18 [1.08, 1.29] | **<0.005** |
