# Supplementary material for: COVID-19 and cardiovascular outcomes in patients with pre-existing hypertension
Source: J Hum Hypertens. 2026 Apr 9;40(6):446–55. doi: 10.1038/s41371-026-01147-4 (PMC13249568; doi:10.1038/s41371-026-01147-4)
Supplement: Supplementary file 5 — Appendix 1 [file 41371_2026_1147_MOESM5_ESM.docx]

The list of antihypertensive medications are as follow: benazepril, captopril, cilazapril, enalapril, enalaprilat, fosinopril, lisinopril, moexipril, perindopril, quinapril, ramipril, trandolapril, azilsartan, candesartan, eprosartan, irbesartan, losartan, olmesartan, telmisartan, valsartan, acebutolol, atenolol, betaxolol, bisoprolol, carteolol, carvedilol, esmolol, labetalol, levobunolol, metropolol, nadolol, nebivolol, pindolol, propranolol, sotalol, timolol, chlorthalidone, hydrochlorothiazide, indapamide, amlodipine, clevidipine, felodipine, flunarizine, isradipine, levamlodipine, nicardipine, nifedipine, nimodipine, nisoldipine, diltiazem, verapamil
