# Supplementary material for: COVID-19 and cardiovascular outcomes in patients with pre-existing hypertension
Source: J Hum Hypertens. 2026 Apr 9;40(6):446–55. doi: 10.1038/s41371-026-01147-4 (PMC13249568; doi:10.1038/s41371-026-01147-4)
Supplement: Supplementary file 6 — Appendix 2 [file 41371_2026_1147_MOESM6_ESM.docx]

Exclusion of patients who died, were lost to follow-up, or experienced MACE during acute infection may have led to selection bias. This analysis here did not exclude patients due to being lost to follow-up or experiencing MACE within the first 30 days of index date. The flowchart is shown in **Figure A**, patients characteristics are shown in **Table A,** and the results are shown in **Table B**.


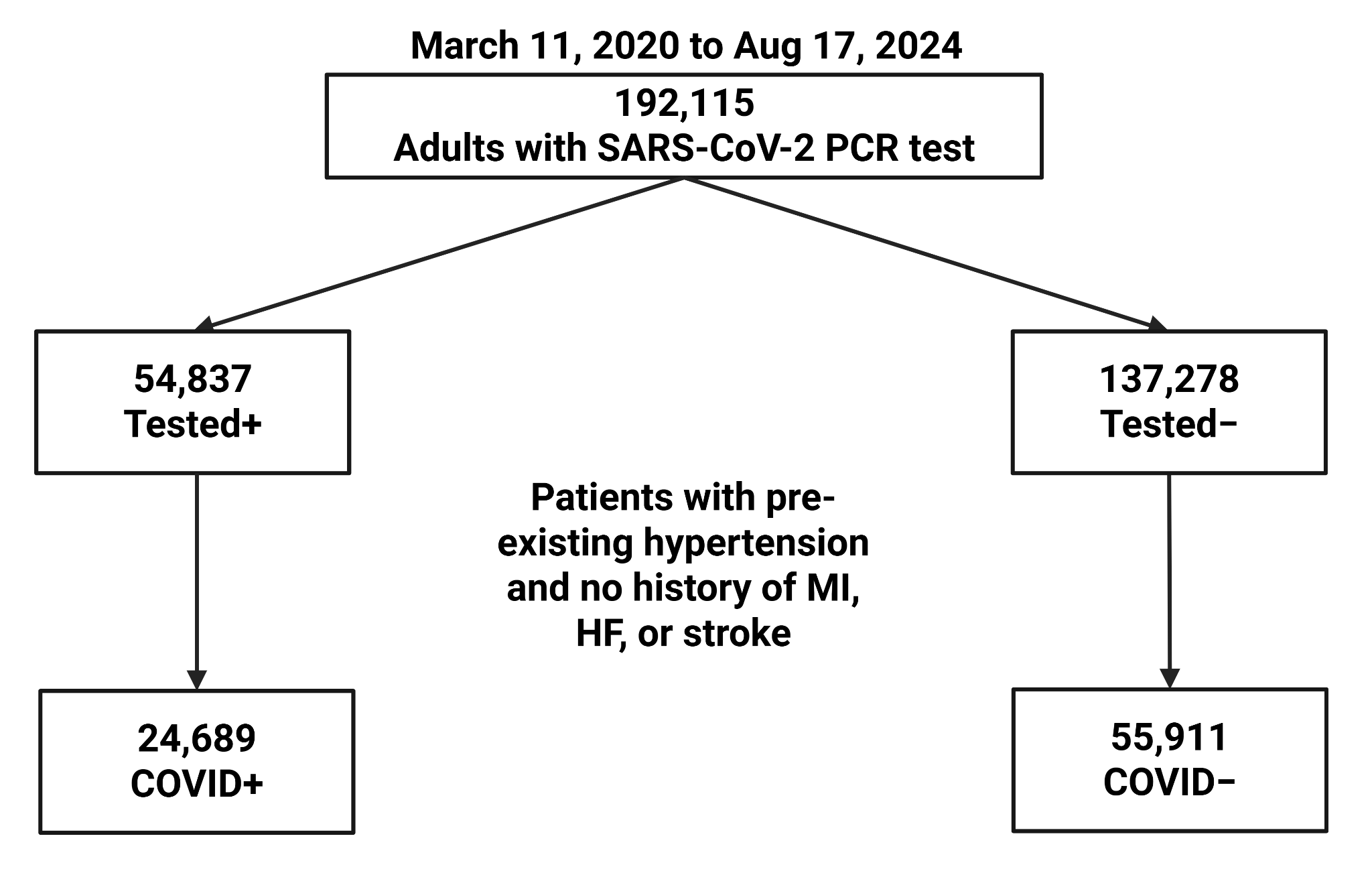


**Figure A.** Patient selection flowchart not excluding patients who experienced outcomes or were lost to follow-up within 30 days of index date. PCR, polymerase chain reaction. MI, myocardial infarction. HF, heart failure.

**Table A.** Characteristics of patients with pre-existing hypertension but no history of myocardial infarction, heart failure, or stroke with and without COVID-19 (no 30-day exclusions applied). SD, standard deviation. SMD, standardized mean difference. COPD, chronic obstructive pulmonary disease.

|  | **COVID+ (n=24689)** | **COVID– (n=55911)** | *p*-value | SMD |
| --- | --- | --- | --- | --- |
| Follow Up Time (Months), mean ± SD | 22.17 ± 14.89 | 26.05 ± 15.05 | **<0.005** | 0.26 |
| Age at Index Date (Years), mean ± SD | 56.30 ± 17.05 | 54.71 ± 15.70 | **<0.005** | 0.097 |
| Female, n (%) | 15470 (62.66%) | 34507 (61.72%) | **0.011** | 0.019 |
| **Race and Ethnicity, n (%)** |  |  |  |  |
| Non-Hispanic White | 2331 (9.44%) | 5770 (10.32%) | **<0.005** | 0.029 |
| Black | 8799 (35.64%) | 19187 (34.32%) | **<0.005** | 0.028 |
| Asian | 1128 (4.57%) | 2269 (4.06%) | **<0.005** | 0.025 |
| Other Race | 12431 (50.35%) | 28685 (51.30%) | **0.013** | 0.019 |
| Hispanic | 10336 (41.86%) | 22926 (41.00%) | **0.023** | 0.017 |
| **Blood Pressure (mm Hg), mean ± SD** |  |  |  |  |
| Systolic Blood Pressure | 133.08 ± 13.91 | 133.92 ± 14.25 | **<0.005** | 0.060 |
| Diastolic Blood Pressure | 78.61 ± 8.48 | 80.05 ± 8.25 | **<0.005** | 0.17 |
| **Stage of Hypertension, n (%)** |  |  |  |  |
| No Blood Pressure Measurements Available | 5163 (20.91%) | 12298 (22.00%) | **<0.005** | 0.026 |
| Normal | 2409 (9.76%) | 3972 (7.10%) | **<0.005** | 0.096 |
| Elevated | 4218 (17.08%) | 8635 (15.44%) | **<0.005** | 0.044 |
| Stage 1 Hypertension | 7206 (29.19%) | 17303 (30.95%) | **<0.005** | 0.038 |
| Stage 2 Hypertension | 5693 (23.06%) | 13703 (24.51%) | **<0.005** | 0.034 |
| **Definition of Hypertension Met, n (%)** |  |  |  |  |
| Blood Pressure Measurements | 17117 (69.33%) | 39641 (70.90%) | **<0.005** | 0.034 |
| Antihypertensive Use | 15115 (61.22%) | 30998 (55.44%) | **<0.005** | 0.12 |
| ICD-10 Code | 17397 (70.46%) | 35020 (62.64%) | **<0.005** | 0.17 |
| **Pre-Existing Comorbidities, n (%)** |  |  |  |  |
| Coronary Artery Disease | 2491 (10.09%) | 3439 (6.15%) | **<0.005** | 0.14 |
| Type-2 Diabetes | 8676 (35.14%) | 14965 (26.77%) | **<0.005** | 0.18 |
| COPD | 1159 (4.69%) | 1256 (2.25%) | **<0.005** | 0.13 |
| Asthma | 5485 (22.22%) | 9466 (16.93%) | **<0.005** | 0.13 |
| Chronic Kidney Disease | 4225 (17.11%) | 5320 (9.52%) | **<0.005** | 0.23 |
| Liver Disease | 2324 (9.41%) | 4119 (7.37%) | **<0.005** | 0.074 |
| Obesity | 14212 (57.56%) | 29775 (53.25%) | **<0.005** | 0.087 |
| Tobacco Use | 8395 (34.00%) | 19529 (34.93%) | **0.011** | 0.019 |
| **Insurance, n (%)** |  |  |  |  |
| Medicaid | 8316 (33.68%) | 20163 (36.06%) | **<0.005** | 0.050 |
| Medicare | 5306 (21.49%) | 9796 (17.52%) | **<0.005** | 0.10 |
| Private | 9206 (37.29%) | 22232 (39.76%) | **<0.005** | 0.051 |
| Uninsured | 1861 (7.54%) | 3720 (6.65%) | **<0.005** | 0.034 |
| **Income Group, n (%)** |  |  |  |  |
| Lower Third (≤$42,639/year) | 9934 (40.24%) | 22766 (40.72%) | 0.20 | 0.0098 |
| Middle Third ($42,834/year–$61,272/year) | 7250 (29.37%) | 15845 (28.34%) | **<0.005** | 0.023 |
| Top Third (≥$61,414/year) | 7505 (30.40%) | 17300 (30.94%) | 0.13 | 0.012 |
| **Unmet Social Needs, n (%)** |  |  |  |  |
| At Least One Unmet Social Need | 2163 (8.76%) | 4322 (7.73%) | **<0.005** | 0.037 |
| No Unmet Social Needs | 6345 (25.70%) | 13633 (24.38%) | **<0.005** | 0.03 |
| Status Unknown | 16181 (65.54%) | 37956 (67.89%) | **<0.005** | 0.05 |
| **Hospitalized Due to COVID-19, n (%)** | 8123 (32.90%) | 0 (0.00%) | **<0.005** | 0.99 |
| **Vaccinated for SARS-CoV-2, n (%)** | 9211 (37.31%) | 16789 (30.03%) | **<0.005** | 0.15 |
| **Outcomes, n (%)** |  |  |  |  |
| All-Cause Mortality | 1377 (5.58%) | 644 (1.15%) | **<0.005** | 0.25 |
| Myocardial Infarction | 515 (2.09%) | 936 (1.67%) | **<0.005** | 0.03 |
| Heart Failure | 1290 (5.22%) | 2177 (3.89%) | **<0.005** | 0.064 |
| Ischemic or Hemorrhagic Stroke | 511 (2.07%) | 966 (1.73%) | **<0.005** | 0.025 |
| Major Adverse Cardiovascular Events | 3124 (12.65%) | 3916 (7.00%) | **<0.005** | 0.19 |

**Table B.** Cox proportional (all-cause mortality and major adverse cardiovascular events) and Fine-Gray subdistribution (myocardial infarction, heart failure, and ischemic or hemorrhagic stroke) adjusted hazard ratios (HR) for different outcomes grouped by COVID-19 status (COVID+ hospitalized and COVID+ non-hospitalized vs. COVID–) (no 30-day exclusions applied). Multivariate model for mortality could not converge due to very high COVID+ hospitalized hazard ratio (HR). Multivariate regression and inverse probability weighting both adjusted for baseline age, sex, race, ethnicity, comorbidities, stage of hypertension, insurance status, tertile of Zone Improvement Plan median income, presence of unmet social needs, and SARS-CoV-2 vaccination status. HR, hazard ratio. CI, confidence interval.

|  | **Multivariate Regression** | | | |
| --- | --- | --- | --- | --- |
| **Outcome** | COVID+ Hospitalized vs COVID– | | COVID+ Non-Hospitalized vs COVID– | |
|  | Adjusted HR [95% CI] | *p*-value | Adjusted HR [95% CI] | *p*-value |
| Myocardial Infarction | 1.41 [1.23, 1.62] | **<0.005** | 1.06 [0.91, 1.22] | 0.48 |
| Heart Failure | 1.56 [1.43, 1.71] | **<0.005** | 1.11 [1.01, 1.22] | **0.025** |
| Ischemic or Hemorrhagic Stroke | 1.39 [1.21, 1.60] | **<0.005** | 1.06 [0.92, 1.23] | 0.41 |
| Major Adverse Cardiovascular Events | 2.45 [2.31, 2.59] | **<0.005** | 1.16 [1.08, 1.24] | **<0.005** |
|  | **Inverse Probability Weighting-Adjusted** | | | |
| **Outcome** | COVID+ Hospitalized vs COVID– | | COVID+ Non-Hospitalized vs COVID– | |
|  | HR [95% CI] | *p*-value | HR [95% CI] | *p*-value |
| All-Cause Mortality | 6.49 [5.80, 7.26] | **<0.005** | 1.72 [1.46, 2.02] | **<0.005** |
| Myocardial Infarction | 1.55 [1.30, 1.85] | **<0.005** | 1.08 [0.92, 1.26] | 0.37 |
| Heart Failure | 1.70 [1.53, 1.90] | **<0.005** | 1.11 [1.00, 1.23] | **0.041** |
| Ischemic or Hemorrhagic Stroke | 1.37 [1.15, 1.64] | **<0.005** | 1.00 [0.86, 1.17] | 0.96 |
| Major Adverse Cardiovascular Events | 2.42 [2.26, 2.61] | **<0.005** | 1.18 [1.09, 1.27] | **<0.005** |
